# Genome-Wide Joint Meta-Analysis of SNP and SNP-by-Smoking Interaction Identifies Novel Loci for Pulmonary Function

**DOI:** 10.1371/journal.pgen.1003098

**Published:** 2012-12-20

**Authors:** Dana B. Hancock, María Soler Artigas, Sina A. Gharib, Amanda Henry, Ani Manichaikul, Adaikalavan Ramasamy, Daan W. Loth, Medea Imboden, Beate Koch, Wendy L. McArdle, Albert V. Smith, Joanna Smolonska, Akshay Sood, Wenbo Tang, Jemma B. Wilk, Guangju Zhai, Jing Hua Zhao, Hugues Aschard, Kristin M. Burkart, Ivan Curjuric, Mark Eijgelsheim, Paul Elliott, Xiangjun Gu, Tamara B. Harris, Christer Janson, Georg Homuth, Pirro G. Hysi, Jason Z. Liu, Laura R. Loehr, Kurt Lohman, Ruth J. F. Loos, Alisa K. Manning, Kristin D. Marciante, Ma'en Obeidat, Dirkje S. Postma, Melinda C. Aldrich, Guy G. Brusselle, Ting-hsu Chen, Gudny Eiriksdottir, Nora Franceschini, Joachim Heinrich, Jerome I. Rotter, Cisca Wijmenga, O. Dale Williams, Amy R. Bentley, Albert Hofman, Cathy C. Laurie, Thomas Lumley, Alanna C. Morrison, Bonnie R. Joubert, Fernando Rivadeneira, David J. Couper, Stephen B. Kritchevsky, Yongmei Liu, Matthias Wjst, Louise V. Wain, Judith M. Vonk, André G. Uitterlinden, Thierry Rochat, Stephen S. Rich, Bruce M. Psaty, George T. O'Connor, Kari E. North, Daniel B. Mirel, Bernd Meibohm, Lenore J. Launer, Kay-Tee Khaw, Anna-Liisa Hartikainen, Christopher J. Hammond, Sven Gläser, Jonathan Marchini, Peter Kraft, Nicholas J. Wareham, Henry Völzke, Bruno H. C. Stricker, Timothy D. Spector, Nicole M. Probst-Hensch, Deborah Jarvis, Marjo-Riitta Jarvelin, Susan R. Heckbert, Vilmundur Gudnason, H. Marike Boezen, R. Graham Barr, Patricia A. Cassano, David P. Strachan, Myriam Fornage, Ian P. Hall, Josée Dupuis, Martin D. Tobin, Stephanie J. London

**Affiliations:** 1 Behavioral Health Epidemiology Program, Research Triangle Institute International, Research Triangle Park, North Carolina, United States of America; 2 Epidemiology Branch, National Institute of Environmental Health Sciences, National Institutes of Health, U.S. Department of Health and Human Services, Research Triangle Park, North Carolina, United States of America; 3 Departments of Health Sciences and Genetics, University of Leicester, Leicester, United Kingdom; 4 Center for Lung Biology, University of Washington, Seattle, Washington, United States of America; 5 Department of Medicine, University of Washington, Seattle, Washington, United States of America; 6 Division of Therapeutics and Molecular Medicine, University of Nottingham, Queen's Medical Centre, Nottingham, United Kingdom; 7 Center for Public Health Genomics, University of Virginia, Charlottesville, Virginia, United States of America; 8 Department of Public Health Sciences, Division of Biostatistics and Epidemiology, University of Virginia, Charlottesville, Virginia, United States of America; 9 Respiratory Epidemiology and Public Health, Imperial College London, London, United Kingdom; 10 Department of Epidemiology and Biostatistics, School of Public Health, Imperial College London, London, United Kingdom; 11 Department of Medical and Molecular Genetics, King's College London, Guy's Hospital, London, United Kingdom; 12 Department of Epidemiology, Erasmus Medical Center, Rotterdam, The Netherlands; 13 Inspectorate of Healthcare, The Hague, The Netherlands; 14 Department of Epidemiology and Public Health, Swiss Tropical and Public Health Institute, Basel, Switzerland; 15 University of Basel, Basel, Switzerland; 16 Department of Internal Medicine B, University Hospital Greifswald, Greifswald, Germany; 17 ALSPAC, School of Social and Community Medicine, University of Bristol, Bristol, United Kingdom; 18 Icelandic Heart Association, Kopavogur, Iceland; 19 University of Iceland, Reykjavik, Iceland; 20 Department of Genetics, University of Groningen, University Medical Center Groningen, Groningen, The Netherlands; 21 Department of Medicine, University of New Mexico, Albuquerque, New Mexico, United States of America; 22 Division of Nutritional Sciences, Cornell University, Ithaca, New York, United States of America; 23 Division of Aging, Department of Medicine, Brigham and Women's Hospital and Harvard Medical School, Boston, Massachusetts, United States of America; 24 The National Heart, Lung, and Blood Institute's Framingham Heart Study, Framingham, Massachusetts, United States of America; 25 Department of Twin Research and Genetic Epidemiology, King's College London, London, United Kingdom; 26 Discipline of Genetics, Faculty of Medicine, Memorial University of Newfoundland, St. John's, Newfoundland, Canada; 27 MRC Epidemiology Unit, Institute of Metabolic Science, Cambridge, United Kingdom; 28 Program in Molecular and Genetic Epidemiology, Harvard School of Public Health, Boston, Massachusetts, United States of America; 29 Department of Medicine, College of Physicians and Surgeons, Columbia University, New York, New York, United States of America; 30 MRC-HPA Centre for Environment and Health, Imperial College London, London, United Kingdom; 31 Brown Foundation Institute of Molecular Medicine, University of Texas Health Science Center at Houston, Houston, Texas, United States of America; 32 National Institute on Aging, National Institutes of Health, Bethesda, Maryland, United States of America; 33 Department of Medical Sciences, Respiratory Medicine, Uppsala University, Uppsala, Sweden; 34 Interfaculty Institute for Genetics and Functional Genomics, Department of Functional Genomics, University of Greifswald, Greifswald, Germany; 35 Department of Statistics, University of Oxford, United Kingdom; 36 Gillings School of Global Public Health, Department of Epidemiology, University of North Carolina at Chapel Hill, Chapel Hill, North Carolina, United States of America; 37 Department of Biostatistical Sciences, Division of Public Health Sciences, Wake Forest School of Medicine, Winston-Salem, North Carolina, United States of America; 38 Program in Medical and Population Genetics, Broad Institute, Cambridge, Massachusetts, United States of America; 39 Department of Molecular Biology, Massachusetts General Hospital, Boston, Massachusetts, United States of America; 40 Department of Genetics, Harvard Medical School, Boston, Massachusetts, United States of America; 41 Department of Pulmonology, University of Groningen, University Medical Center Groningen, Groningen, The Netherlands; 42 GRIAC Research Institute, University Medical Center Groningen, Groningen, The Netherlands; 43 Department of Thoracic Surgery and Division of Epidemiology, Vanderbilt University Medical Center, Nashville, Tennessee, United States of America; 44 Department of Respiratory Medicine, Ghent University Hospital, Ghent, Belgium; 45 Section of Pulmonary and Critical Care Medicine, Department of Medicine, Veterans Administration Boston Healthcare System, Boston, Massachusetts, United States of America; 46 The Pulmonary Center, Department of Medicine, Boston University School of Medicine, Boston, Massachusetts, United States of America; 47 Institute of Epidemiology I, Helmholtz Zentrum München, Munich, Germany; 48 Medical Genetics Institute, Cedars-Sinai Medical Center, Los Angeles, California, United States of America; 49 Department of Biostatistics, Robert Stempel College of Public Health and Social Work, Florida International University, Miami, Florida, United States of America; 50 Center for Research on Genomics and Global Health, National Human Genome Research Institute, National Institutes of Health, Bethesda, Maryland, United States of America; 51 Department of Biostatistics, University of Washington, Seattle, Washington, United States of America; 52 Department of Statistics, University of Auckland, Auckland, New Zealand; 53 Human Genetics Center, School of Public Health, University of Texas Health Science Center at Houston, Houston, Texas, United States of America; 54 Department of Internal Medicine, Erasmus Medical Center, Rotterdam, The Netherlands; 55 Netherlands Genomics Initiative (NGI)–sponsored Netherlands Consortium for Healthy Aging (NCHA), Leiden, The Netherlands; 56 Department of Biostatistics, University of North Carolina at Chapel Hill, Chapel Hill, North Carolina, United States of America; 57 Sticht Center on Aging, Wake Forest School of Medicine, Winston-Salem, North Carolina, United States of America; 58 Department of Epidemiology and Prevention, Division of Public Health Sciences, Wake Forest School of Medicine, Winston-Salem, North Carolina, United States of America; 59 Institute of Lung Biology and Disease, Comprehensive Pneumology Center, Helmholtz Zentrum München, Neuherberg, Germany; 60 Institute for Medical Statistics and Epidemiology (IMSE), Technical University Munich, Munich, Germany; 61 Department of Epidemiology, University of Groningen, University Medical Center Groningen, Groningen, The Netherlands; 62 The LifeLines Cohort Study, Groningen, The Netherlands; 63 Division of Pulmonary Medicine, University Hospitals of Geneva, Geneva, Switzerland; 64 Cardiovascular Health Research Unit and Department of Epidemiology, University of Washington, Seattle, Washington, United States of America; 65 Departments of Medicine and Health Services, University of Washington, Seattle, United States of America; 66 Group Health Research Institute, Group Health Cooperative, Seattle, Washington; 67 Broad Institute of MIT and Harvard, Cambridge, Massachusetts, United States of America; 68 College of Pharmacy, University of Tennessee Health Science Center, Memphis, Tennessee, United States of America; 69 Department of Public Health and Primary Care, University of Cambridge, Cambridge, United Kingdom; 70 Department of Obstetrics and Gynecology, Institute of Clinical Medicine, University of Oulu, Oulu, Finland; 71 Departments of Epidemiology and Biostatistics, Harvard School of Public Health, Boston, Massachusetts, United States of America; 72 Institute for Community Medicine, Study of Health In Pomerania (SHIP)/Clinical Epidemiological Research, University of Greifswald, Greifswald, Germany; 73 Department of Medical Informatics, Erasmus Medical Center, Rotterdam, The Netherlands; 74 Department of Children, Young People, and Families, National Institute for Health and Welfare, Oulu, Finland; 75 Institute of Health Sciences, University of Oulu, Oulu, Finland; 76 Biocenter Oulu, University of Oulu, Oulu, Finland; 77 Department of Epidemiology, Mailman School of Public Health, Columbia University, New York, New York, United States of America; 78 Department of Public Health, Weill Cornell Medical College, New York, New York, United States of America; 79 Division of Population Health Sciences and Education, St. George's University of London, London, United Kingdom; 80 Department of Biostatistics, Boston University School of Public Health, Boston, Massachusetts, United States of America; 81 Laboratory of Respiratory Biology, National Institute of Environmental Health Sciences, National Institutes of Health, U.S. Department of Health and Human Services, Research Triangle Park, North Carolina, United States of America; Georgia Institute of Technology, United States of America

## Abstract

Genome-wide association studies have identified numerous genetic loci for spirometic measures of pulmonary function, forced expiratory volume in one second (FEV_1_), and its ratio to forced vital capacity (FEV_1_/FVC). Given that cigarette smoking adversely affects pulmonary function, we conducted genome-wide joint meta-analyses (JMA) of single nucleotide polymorphism (SNP) and SNP-by-smoking (ever-smoking or pack-years) associations on FEV_1_ and FEV_1_/FVC across 19 studies (total N = 50,047). We identified three novel loci not previously associated with pulmonary function. SNPs in or near *DNER* (smallest *P*_JMA = _5.00×10^−11^), *HLA-DQB1* and *HLA-DQA2* (smallest *P*_JMA = _4.35×10^−9^), and *KCNJ2* and *SOX9* (smallest *P*_JMA = _1.28×10^−8^) were associated with FEV_1_/FVC or FEV_1_ in meta-analysis models including SNP main effects, smoking main effects, and SNP-by-smoking (ever-smoking or pack-years) interaction. The HLA region has been widely implicated for autoimmune and lung phenotypes, unlike the other novel loci, which have not been widely implicated. We evaluated *DNER*, *KCNJ2*, and *SOX9* and found them to be expressed in human lung tissue. *DNER* and *SOX9* further showed evidence of differential expression in human airway epithelium in smokers compared to non-smokers. Our findings demonstrated that joint testing of SNP and SNP-by-environment interaction identified novel loci associated with complex traits that are missed when considering only the genetic main effects.

## Author Summary

Measures of pulmonary function provide important clinical tools for evaluating lung disease and its progression. Genome-wide association studies have identified numerous genetic risk factors for pulmonary function but have not considered interaction with cigarette smoking, which has consistently been shown to adversely impact pulmonary function. In over 50,000 study participants of European descent, we applied a recently developed joint meta-analysis method to simultaneously test associations of gene and gene-by-smoking interactions in relation to two major clinical measures of pulmonary function. Using this joint method to incorporate genetic main effects plus gene-by-smoking interaction, we identified three novel gene regions not previously related to pulmonary function: (1) *DNER*, (2) *HLA-DQB1* and *HLA-DQA2*, and (3) *KCNJ2* and *SOX9*. Expression analyses in human lung tissue from ours or prior studies indicate that these regions contain genes that are plausibly involved in pulmonary function. This work highlights the utility of employing novel methods for incorporating environmental interaction in genome-wide association studies to identify novel genetic regions.

## Introduction

Spirometric measures of pulmonary function, particularly forced expiratory volume in one second (FEV_1_) and its ratio to forced vital capacity (FEV_1_/FVC), are important clinical tools for diagnosing pulmonary disease, classifying its severity, and evaluating its progression over time. These measures also predict other morbidities and mortality in the general population [1]–[3]. Genetic factors likely play a prominent role in determining the maximal level of pulmonary function in early adulthood and its subsequent decline with age [4], [5]. A relatively uncommon deficiency of α-1 antitrypsin, due to homozygous mutations of the *SERPINA1* gene, is a well-established genetic risk factor for accelerated decline in pulmonary function, but it accounts for little of the population variability in pulmonary function.

Genome-wide association studies (GWAS) have identified many common genetic variants underlying pulmonary function. The first GWAS of pulmonary function implicated *HHIP* for FEV_1_/FVC [6], [7]. GWAS meta-analyses for FEV_1_/FVC and FEV_1_ from the Cohorts for Heart and Aging Research in Genomic Epidemiology (CHARGE) and SpiroMeta Consortia have together identified 26 additional novel loci in or near the following genes: *ADAM19*, *AGER-PPT2*, *ARMC2*, *C10orf11*, *CCDC38*, *CDC123*, *CFDP1*, *FAM13A*, *GPR126*, *HDAC4*, *HTR*4, *INTS12-GSTCD-NPNT*, *KCNE2*, *LRP1*, *MECOM* (*EVI1*), *MFAP2*, *MMP15*, *NCR3*, *PID1*, *PTCH1*, *RARB*, *SPATA9*, *TGFB2*, *THSD4*, *TNS1*, and *ZKSCAN3* [8]–[10].

Inhaled pollutants, especially cigarette smoking, can have important adverse effects on pulmonary function. Candidate gene studies have not consistently identified interactions with cigarette smoking in relation to pulmonary function. Despite the importance of smoking and other environmental factors in the etiology of many complex human diseases and traits, few GWAS have incorporated gene-by-environment interactions [11]–[14]. Meta-analyses are generally necessary to provide sufficient sample size to detect moderate effects, and methods for joint testing of single nucleotide polymorphism (SNP) main effects and SNP-by-environment interactions in the meta-analysis setting have only recently been developed [15], [16]. This strategy has the potential to identify novel loci that would not emerge from analyses based on the SNP main or interactive effects alone [15]–[17]. The well-documented and consistent deleterious effect of cigarette smoking on pulmonary function [18] makes it a good candidate for such an approach, since genetic factors may have heterogeneous effects on pulmonary function depending on smoking exposure. We conducted genome-wide joint meta-analyses (JMA) of SNP and SNP-by-smoking interaction (ever-smoking or pack-years) associations with cross-sectional pulmonary function measures (FEV_1_/FVC and FEV_1_) in 50,047 study participants of European ancestry.

## Results

Table S1 presents characteristics of the 50,047 participants from 19 studies contributing to our analyses. As expected, mean FEV_1_ and FVC values were lower in studies with the oldest participants. Standardized residuals of FEV_1_ and FEV_1_/FVC (see Methods) were used as the phenotypes for the JMA, in order to maximize comparability with our recent GWAS meta-analysis from the CHARGE and SpiroMeta Consortia [10]. Our original GWAS meta-analyses, conducted separately in CHARGE and SpiroMeta, showed that we were able to identify replicable genetic loci whether using actual pulmonary function measures [8] or their standardized residuals [9]. The standardized residual approach was similarly taken in GWAS of other complex quantitative traits, such as height and body mass index from the Genetic Investigation of ANthropometric Traits (GIANT) Consortium [19], [20].

In each of the 19 studies, four regression models with differing SNP-by-smoking interaction terms were run: (1) SNP-by-ever-smoking for standardized FEV_1_/FVC residuals, (2) SNP-by-pack-years for standardized FEV_1_/FVC residuals, (3) SNP-by-ever-smoking for standardized FEV_1_ residuals, and (4) SNP-by-pack-years for standardized FEV_1_ residuals. Study-specific genomic inflation factors (λ_gc_) were calculated for the 1 degree-of-freedom (d.f.) SNP-by-smoking interaction term, to ensure that there was no substantial inflation due to the main effect of smoking being misspecified [21]. All study-specific results had 1 d.f. λ_gc_≤1.09 (Table S2), which is of comparable magnitude to other studies with large sample sizes [10], [19], [22], [23].

The study-specific regression coefficients from each of the four models were then combined in JMA, and the resulting λ_gc_ values from the 2 d.f. JMA, calculated across all SNPs, ranged from 1.056 to 1.064. The quantile-quantile plots (Figure S1) show substantial deviation from expectation for SNPs having low *P* values from the JMA (*P*_JMA_). The JMA results corresponding to the top SNP from each previously implicated locus [8]–[10] are presented in Table S3. To identify novel loci among the genome-wide significant loci implicated by our JMA models, the genomic regions surrounding the most significant SNP from each of the 27 previously implicated loci [8]–[10] (500 kb upstream to 500 kb downstream of each SNP) were removed from consideration (Table S3). Following the removal of all previously implicated loci [8]–[10], the quantile-quantile plots show that some deviation remained between observed and expected *P* values for high-signal SNPs suggesting the presence of novel signals.

**Figure 1:**
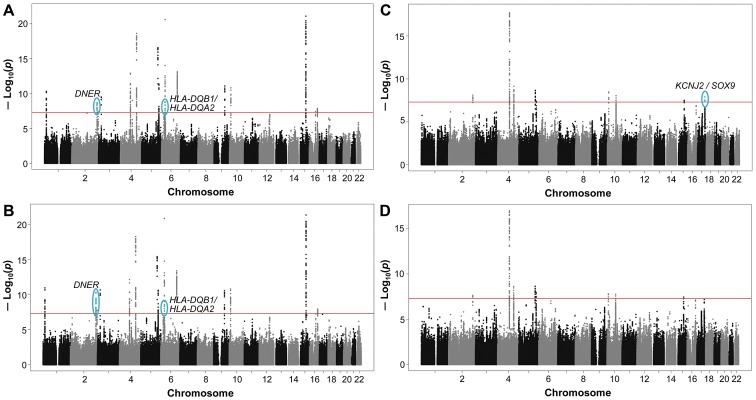
Genome-wide joint meta-analysis (JMA) of SNP and SNP-by-smoking interaction in relation to pulmonary function. The Manhattan plots show the chromosomal position of SNPs in comparison to their −log_10_ *P*_JMA_ values. JMA results are shown for models with (A) SNP-by-ever-smoking interaction term in relation to FEV_1_/FVC, (B) SNP-by-pack-years interaction term in relation to FEV_1_/FVC, (C) SNP-by-ever-smoking interaction term in relation to FEV_1_, and (D) SNP-by-pack-years interaction term in relation to FEV_1_. SNPs located within previously implicated loci are shown, but these loci were not considered when identifying novel loci from the joint modeling of SNP main effects and smoking interactive effects. Novel loci on chromosomes 2, 6, and 17 (shown in blue and circled) were identified as those having SNPs with genome-wide significant *P* values at the standard threshold (*P*<5×10^−8^ as indicated by the solid red line). Names of the novel gene (or closest genes) are provided.

In the JMA of SNP and SNP-by-smoking in relation to FEV_1_/FVC, we observed two novel loci containing several significant SNP associations at the standard genome-wide Bonferroni-corrected threshold of *P*_JMA_<5×10^−8^, when considering interaction with ever-smoking (Figure 1A) or pack-years (Figure 1B). The SNP associations from both loci also exceeded the more conservative genome-wide significance threshold of *P*_JMA_<1.25×10^−8^, based on additional Bonferroni correction for the four JMA models.

**Figure 2:**
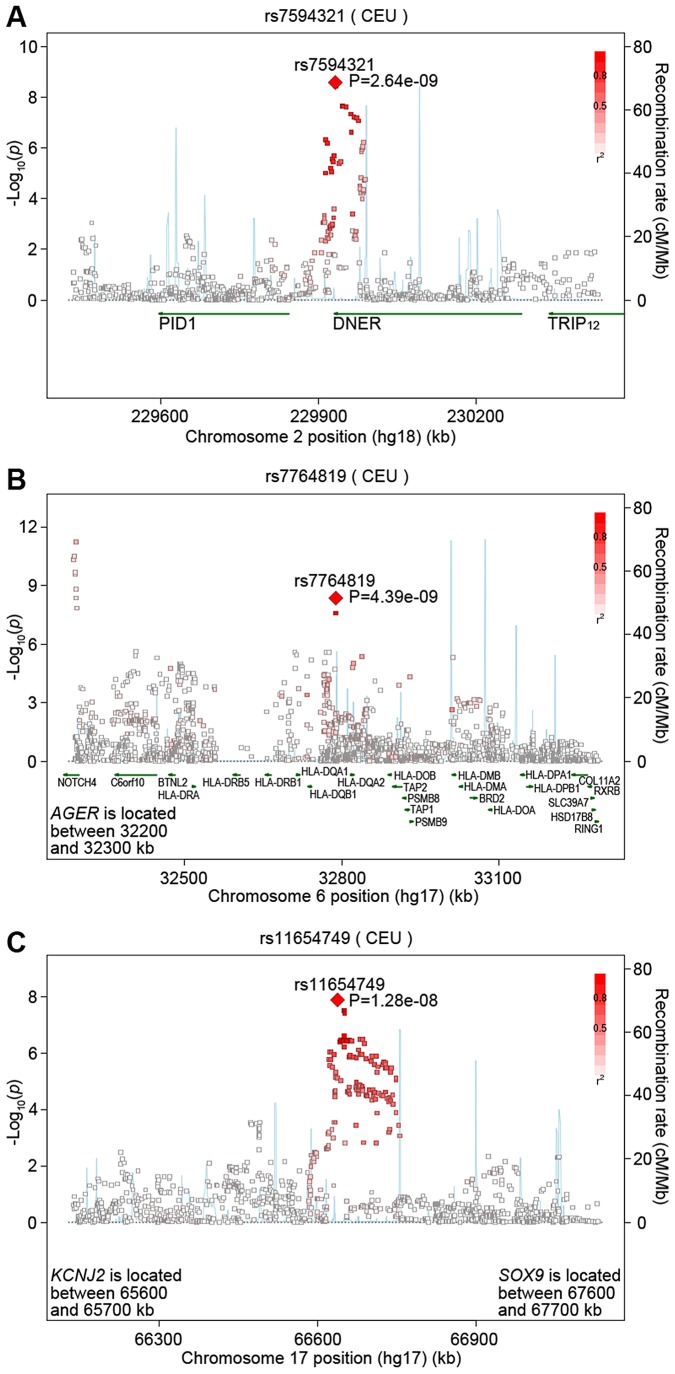
Regional association plots of novel loci implicated for pulmonary function. Three novel loci contained SNPs associated with FEV_1_/FVC or FEV_1_ at the standard genome-wide significance threshold (*P*<5×10^−8^) in joint meta-analyses of SNP and SNP-by-smoking interaction. SNPs are shown within 500 kb of the most significant SNPs on chromosomes (A) 2q36.3 associated with FEV_1_/FVC, (B) 6p21.32 associated with FEV_1_/FVC, and (C) 17q24.3 associated with FEV_1_. Pairwise r^2^ values were based on the HapMap CEU population, and progressively darker shades of red indicate higher r^2^ values. Estimated recombination rates from HapMap are shown as background lines.

The most statistically significant result was for rs7594321, an intronic SNP located in *DNER* (delta/notch-like EGF-related receptor) on chromosome 2, which gave *P*_JMA_ = 2.64×10^−9^ (corresponding *P*_INT_ = 0.27) in the ever-smoking model and *P*_JMA_ = 5.00×10^−11^ (corresponding *P*_INT_ = 0.0069) in the pack-years model (Table 1). For the ever/never-smoking interaction model, the observed level of significance for the JMA is plausible in the presence of a nominally significant SNP main effect and a nonsignificant interactive effect, as detailed in Text S1. The rs7594321 T allele had a positive β coefficient for the genetic main association and a negative β coefficient for the interaction (Table 1, Table S4 for study-specific results). The regression coefficients correspond to a per allele change of 0.049 (95% CI: 0.030, 0.068) in never-smokers and 0.035 (95% CI: 0.016, 0.053) in ever-smokers. A conserved binding site for the Zic1 transcription factor is located 115 base pairs away from rs7594321. Further, rs7594321 is located upstream of the previously implicated *PID1* gene (Figure 2A), but it is 713 kb away from the previously implicated SNP (rs1435867), which is located downstream of *PID1*. There is no linkage disequilibrium (LD) between rs7594321 and rs1435867 (r^2^ = 0, D′ = 0).

**Table 1:** Genome-wide significant SNPs from the joint meta-analysis (JMA) of SNP and SNP-by-smoking (ever-smoking or pack-years) interaction in relation to pulmonary function.

SNP (coded allele)	Chr	Gene/closest gene(s)	Coded allele frequency^1^	JMA results							
				Smoking metric	β_SNP_ 2	SE_SNP_	*P*_SNP_	β_INT_ 3	SE_INT_	*P*_INT_	*P*_JMA_
***SNPs implicated in relation to FEV_1_/FVC***											
rs7594321 (T)	2q36.3	*DNER*	0.35	Ever-smoking	0.049	0.0097	4.14×10^−7^	−0.015	0.013	0.27	2.64×10^−9^
				Pack-years	0.048	0.0070	7.03×10^−12^	−0.00020	0.000074	6.88×10^−3^	5.00×10^−11^
rs7764819 (T)	6p21.32	*HLA-DQB1/HLA-DQA2*	0.89	Ever-smoking	−0.060	0.015	6.32×10^−5^	−0.0010	0.021	0.63	4.39×10^−9^
				Pack-years	−0.064	0.011	5.95×10^−9^	−0.000058	0.00010	0.56	4.35×10^−9^
***SNPs implicated in relation to FEV_1_***											
rs11654749 (T)	17q24.3	*KCNJ2/SOX9*	0.39	Ever-smoking	−0.028	0.0094	2.46×10^−3^	−0.017	0.013	0.17	1.28×10^−8^
				Pack-years	−0.038	0.0068	2.29×10^−8^	0.000047	0.000068	0.49	6.63×10^−8^

After removing SNPs with known associations with FEV_1_/FVC or FEV_1_, three novel loci with genome-wide significant SNPs (standard threshold of *P*<5×10^−8^) remained from the JMA testing in the current study. The most significant SNP from each locus is shown.

FEV_1_, forced expiratory volume in the first second; FVC, forced vital capacity; JMA, joint meta-analysis; SE, standard error ; SNP, single nucleotide polymorphism.

1 Weighted average coded allele frequency across the 19 studies. The coded allele refers to the effect allele.

2 β_SNP_, per allele change in the FEV_1_/FVC standardized residual due to the SNP main association.

3 β_INT_, per allele change in the FEV_1_/FVC standardized residual due to the interaction between SNP and smoking.

Our next most statistically significant SNP (rs7764819) is intergenic between two human leukocyte antigen (HLA) genes, *HLA-DQB1* and *HLA-DQA2*, on chromosome 6 (Figure 2B). The *HLA-DQ* region is highly variable, and the association signal in this region is largely driven by two SNPs that are in high LD with one another (rs7764819 and rs7765379, r^2^ = 1) but only low to moderate LD with all other genotyped and imputed SNPs. A GWAS meta-analysis of asthma implicating the *HLA-DQ* region similarly found highly significant associations with only a few SNPs [24]. Our top SNP rs7764819 gave *P*_JMA_ = 4.39×10^−9^ in the ever-smoking model and *P*_JMA_ = 4.35×10^−9^ in the pack-years model for FEV_1_/FVC (Table 1). The corresponding *P*_INT_ values were >0.05 (see Text S1). The rs7764819 T allele had negative β coefficients for both the main association and interaction (Table 1, Table S5 for study-specific results), which correspond to a SNP effect of −0.060 (95% CI: −0.09, −0.031) in never-smokers and −0.070 (95% CI: −0.10, −0.042) in ever-smokers. Although rs7764819 is located 529 kb away from a previously implicated *AGER* SNP (rs2070600), there is some LD between the two SNPs (r^2^ = 0.29, D′ = 0.81). Conserved binding sites for two transcription factors, HTF and Lmo2, are located within 100 kb of rs7764819.

Besides the *DNER* and *HLA-DQB1/HLA-DQA2* loci, SNPs from 12 other chromosomal regions having *P*_JMA_ values between 5×10^−8^ and 1×10^−6^ from either smoking model in relation to FEV_1_/FVC are presented in Table S6. Secondary meta-analyses of the interaction product terms alone identified no SNP-by-smoking (ever-smoking or pack-years) interactions at genome-wide statistical significance with FEV_1_/FVC. SNPs from two chromosomal regions had *P*_INT_ values between 5×10^−8^ and 1×10^−6^ in relation to FEV_1_/FVC, as shown in Table S7.

For FEV_1_, the JMA of SNP and SNP-by-smoking gave genome-wide significant associations (*P*_JMA_<5×10^−8^) in the ever-smoking model for four SNPs on chromosome 17 (Figure 1C). However, these SNP associations did not exceed the more conservative significance threshold of *P*_JMA_<1.25×10^−8^. No novel loci reached genome-wide significance level in the pack-years model in relation to FEV_1_ (Figure 1D).

The most significant SNP (rs11654749) from both smoking models is intergenic between *KCNJ2* (a potassium inwardly-rectifying channel also known as KIR2.1) and *SOX9* (sex determining region Y-box 9) (Figure 2C). Conserved binding sites for four transcription factors (HNF-1, CP2, Cdc5, and FOXF2) are located within 100 kb upstream or downstream of rs11654749. The rs11654749 SNP gave *P*_JMA_ = 1.28×10^−8^ in the ever-smoking model and *P*_JMA_ = 6.63×10^−8^ in the pack-years model (Table 1). The corresponding *P*_INT_ values were >0.05 (see Text S1). The rs11654749 T allele had negative β coefficients for both the main association and interaction (Table 1, Table S8 for study-specific results). These estimates correspond to a SNP effect of −0.028 (95% CI: −0.047, −0.010) in never-smokers and −0.046 (95% CI: −0.063, −0.029) in ever-smokers. To better understand the magnitude of these β estimates, we compared our results with those observed in one of our previous GWAS meta-analyses of SNP main effects [9], where standardized residuals of the pulmonary function measures were similarly computed. For a SNP with MAF around 40%, an absolute β value of 0.028 would be equivalent to 19 mL per copy of the risk allele (comparable to a year of FEV_1_ decline in healthy never-smokers), and an absolute β value of 0.046 would be equivalent to 31 mL per copy of the risk allele (comparable to a year and a half of FEV_1_ decline in healthy never-smokers) [25].

Besides this *KCNJ2*/*SOX9* locus, SNPs from five other chromosomal regions have *P*_JMA_ values between 5×10^−8^ and 1×10^−6^ from either smoking model in relation to FEV_1_ as shown in Table S6. In secondary meta-analyses of the interaction product terms, there were no SNP-by-smoking (ever-smoking or pack-years) interactions implicated at genome-wide statistical significance with FEV_1._ SNPs from four chromosomal regions had *P*_INT_ values between 5×10^−8^ and 1×10^−6^ in relation to FEV_1_, as shown in Table S7.

None of the most significant SNPs from the three novel loci we identified by the JMA were associated with FEV_1_/FVC or FEV_1_ at or near genome-wide significance in our previous GWAS meta-analysis of 48,201 participants from the CHARGE and SpiroMeta Consortia. In fact, the lowest *P* value observed for these SNPs was 1.04×10^−5^ (Table 2) [10].

**Table 2:** Look-up evaluation of SNP main associations with FEV_1_/FVC and FEV_1_ using data generated by our previous genome-wide association study meta-analysis (N = 48,201), for the most significant SNP from each of the three novel loci implicated at genome-wide significance in the joint meta-analysis.

SNP (coded allele)	Gene/closest gene(s)	FEV_1_/FVC			FEV_1_		
		β^1^	SE	*P*	β^1^	SE	*P*
rs7594321 (T)	*DNER*	0.032	0.0072	1.04×10^−5^	0.0081	0.0074	0.27
rs7764819 (T)	*HLA-DQB1/HLA-DQA2*	−0.044	0.011	8.79×10^−5^	−0.0073	0.011	0.52
rs11654749 (T)	*KCNJ2/SOX9*	−0.023	0.0071	0.0015	−0.031	0.0072	1.23×10^−5^

FEV_1_, forced expiratory volume in the first second; FVC, forced vital capacity; SE, standard error; SNP, single nucleotide polymorphism.

1 β_SNP_, per allele change in the FEV_1_/FVC standardized residual due to the SNP main association.

To evaluate whether the three novel loci identified by the JMA were related to smoking, we evaluated their SNP associations with ever-smoking and cigarettes per day using GWAS meta-analysis results from the Oxford-GlaxoSmithKline (Ox-GSK) Consortium (N = 41,150) [26]. None of our implicated SNPs were associated with these smoking phenotypes at *P*<0.05 (Table S9), adding confidence that our JMA-implicated SNP associations were not simply reflective of smoking main effects.

### Expression analyses

Three genes (*DNER*, *KCNJ2*, and *SOX9*) harboring or flanking novel genome-wide significant SNPs were selected for follow-up mRNA expression profiling in human lung tissue and a series of primary cells. Transcripts of all three genes were found in lung tissue, airway smooth muscle, and bronchial epithelial cells; *DNER* and *KCNJ2* transcripts were also found in peripheral blood cells (Table S10).

In a separate line of investigation, using the publically available Gene Expression Omnibus repository [27], [28], we found that the expression profiling of *DNER* and *SOX9* showed differential expression in human airway epithelium of smokers compared to non-smokers (Figure S2A and S2B) [29]. Expression profiling of *KCNJ2* did not show statistically significant differential expression by smoking status (Figure S2C) [29]. We also identified novel genome-wide significant SNPs in the *HLA-DQ* region, but we did not examine *HLA-DQ* expression given the known expression of class II MHC antigens on a range of airway cell types [30], [31]. However, the lead SNP in this region (rs7764819) was associated with statistically significant effects on *HLA-DQB1* expression (*P* = 1.2×10^−14^), according to an eQTL analysis database of lymphoblastoid cell lines [32].

## Discussion

Few GWAS have accounted for potential interaction with environmental risk factors. To identify novel genetic risk factors that are missed when considering only genetic main effects [33], we used the newly available JMA method [15] to simultaneously summarize regression coefficients for the main SNP and SNP-by-smoking interactive effects in 50,047 participants from 19 studies, based on models that were fully saturated for the main effect of smoking. This study represents the most comprehensive analysis to date of gene-by-smoking interaction in relation to pulmonary function. We identified two novel loci (*DNER* and *HLA-DQB1*/*HLA-DQA2*) having highly significant evidence for association with FEV_1_/FVC. A third novel locus (*KCNJ2*/*SOX9*) was associated with FEV_1_. For the most significant SNPs at each of these three loci, there was no evidence for heterogeneity across the studies (smallest heterogeneity *P* = 0.59), indicating that the associations were not driven by one or a few studies and thus reflect accumulation of evidence across the studies. None of these three loci had previously been associated with pulmonary function. The comparison of results with our prior GWAS meta-analysis of SNP main effects [10], using a comparable sample size, suggested that the SNP associations for our top SNPs were weaker in our previous analyses that examined only genetic main effects. However, our analyses and those of Manning et al. [14] suggest that some of the benefit of using the joint test for some findings comes from the careful adjustment for the environmental main effect. Thus, future studies aimed at replicating these findings may wish to jointly test the SNP main and interactive effects [15], [16], [33] instead of implementing a standard test of only the SNP main effects. If there is no evidence for interaction at a given locus, the saturation of the main effect of the environmental factor may be important. The joint testing is applicable for both candidate gene [15] and genome-wide [14] approaches. Further, there was minimal overlap in the top SNPs associated with FEV_1_/FVC and FEV_1_, as similarly observed in our previous GWAS meta-analyses of SNP main effects [8]–[10]. Given that the biological underpinnings of these discrepant association findings remain unknown, future studies should evaluate these genetic loci in the context of the pulmonary function measure for which they were originally implicated.

Given that pulmonary function is a phenotype for which numerous genetic loci have been identified in GWAS and smoking is clearly associated with pulmonary function, it might seem surprising that none of the genome-wide significant SNPs implicated by the JMA demonstrated a substantial interaction *per se*. The lack of strong interactive effects does not negate the well-established harmful effects of cigarette smoking nor the need for broad public health campaigns to curb smoking. Instead, our findings demonstrate the value of applying the newly developed joint methods to uncover novel genetic risk factors that might shed light on the mechanisms leading to reduced pulmonary function.

Our pattern of SNP main and interactive results resemble the patterns seen in another recent application of the same JMA method to incorporate the interaction with body mass index (BMI) into GWAS of type 2 diabetes traits (fasting insulin and blood glucose) [14]. In that study with a sample size of 96,453, nearly double that of ours, the top JMA finding had a corresponding interaction *P* value of 1.6×10^−4^ [14]. In our study, the smallest interaction *P* value for our top JMA finding was 6.9×10^−3^. In both our GWAS of smoking and pulmonary function and the recent GWAS of BMI and diabetes traits [14], the SNPs newly implicated by the JMA had marginally significant associations with the trait under study in models with no interaction term, but they became genome-wide significant when accounting for the environmental factor (cigarette smoking or BMI) and the SNP-by-environment interaction. Our JMA included careful modeling of the environmental factor to saturate the environmental main effects along with the interaction testing. In the GWAS of diabetes traits [14], the careful modeling of the environmental factor appeared to account for some of the novel findings from the JMA, consistent with the modest evidence for interaction [14]. Although our previous GWAS meta-analysis was conducted in ever/never-smoking strata, the regression models were not adjusted for smoking status or pack-years [10]. Some of our novel JMA findings compared with our previous GWAS findings may reflect, in part, the saturated modeling of the smoking main effect rather than the interaction *per se*.

The current analysis of 50,047 participants included only 1,846 more participants than our previous GWAS meta-analysis of SNP main effects [10]. To evaluate the likelihood that this 3.8% increase in sample size above that in our previous meta-analysis of pulmonary function was sufficient to explain our identification of these three novel loci at genome-wide statistical significance in the current JMA, we calculated the statistical power to detect genetic main associations (QUANTO [34]) with minor allele frequency (MAF) and β estimates comparable to the three genome-wide significant SNPs presented in Table 1. The current study (total N = 50,047 participants) had only 0.7% to 4.2% more statistical power than our previous GWAS meta-analysis (total N = 48,201 participants) [10], suggesting that the JMA-implicated SNPs are not merely reflective of increased power to detect genetic main effects. Instead, our novel JMA findings demonstrate an advantage of the method used to jointly test the SNP and SNP-by-smoking interactive effects, including the benefit of the saturated modeling of the smoking main effect.

SNPs located in the *DNER* gene were significantly associated with FEV_1_/FVC, even at the more conservative *P* value threshold of 1.25×10^−8^. The JMA results for *DNER* SNPs were driven by both smoking-adjusted main effects and interaction with quantitative smoking history. The DNER protein product is a ligand of the Notch signaling pathway that has been implicated in neuronal differentiation and maturation [35], [36], adipogenesis [37], and hair-cell development [38]. The Notch pathway is a critical controller of cellular differentiation in multiple organs including the lung [39], [40]. Interestingly, the expression levels of many members of the Notch signaling cascade are significantly altered in airway epithelial cells of smokers [41]. We confirmed the expression of DNER transcripts in lung and peripheral cells, and by mining publicly available transcriptional profiling databases [29], we found that *DNER* is expressed in bronchial epithelial cells of non-smoking adults and, importantly, its expression is significantly higher in smokers (Figure S2A). Collectively, these results suggest that *DNER* plays a role in cigarette smoke-induced airflow obstruction and further corroborate the importance of the Notch signaling circuitry in the pathogenesis of obstructive lung disease.

Also in relation to FEV_1_/FVC, intergenic SNPs between *HLA-DQB1* and *HLA-DQA2* exceeded the more conservative genome-wide significance threshold. The eQTL analyses indicated that the lead SNP is associated with expression of *HLA-DQB1* specifically. However, the major histocompatibility complex region is highly polymorphic with complex LD patterns, and a few specific functional SNPs might explain the observed associations [42]. Genetic variations within this region have been associated with several autoimmune disorders [43] and asthma [24], [44], [45], and an interaction between HLA variants and cigarette smoking has been previously implicated [46]. We found little evidence for interaction with smoking at this locus, suggesting that the JMA results were primarily driven by smoking-adjusted genetic main effects. It is most likely that this locus was not identified in our previous GWAS meta-analysis, because the genetic main associations were not evaluated with careful adjustment for smoking status and pack-years. Adjustment for smoking in the current analysis may have removed residual variance in the outcome that is not attributable to genetic variation [14], thus making the identification of the newly associated SNPs possible.

Intergenic SNPs between *KCNJ2* and *SOX9* were significantly associated with FEV_1_ at the standard *P* value threshold, but not the more conservative threshold. Similar to the HLA region, it appears that the JMA results for the *KCNJ2*/*SOX9* region were primarily driven by smoking-adjusted genetic main effects. This region is enriched for long-range regulatory elements for *SOX9*, although the possibility of this region containing *KCNJ2* regulatory elements cannot be discounted [47]. KCNJ2 is a member of the inwardly-rectifying potassium channel family, which regulates membrane potential and cell excitability and is expressed in many tissues including myocardium, neurons, and vasculature. This potassium channel also affects human bronchial smooth muscle tone and airflow limitation [48]. Dominant negative mutations in *KCNJ2* cause the Andersen syndrome, characterized by ventricular arrhythmias, periodic paralysis, and a number of skeletal and cardiac abnormalities [49]. SOX9 is a transcription factor that is essential for cartilage formation, [50] but it is also abundantly expressed in other tissues including the respiratory epithelium during development [51]. *Sox9^−/−^* and *Sox9^+/−^* mice have multiple skeletal anomalies and severe tracheal cartilage malformations and die prematurely from respiratory insufficiency [50], [52]. Mutations in *SOX9* cause campomelic dysplasia characterized by skeletal defects and autosomal sex reversal [53]. These individuals develop respiratory distress due to chest wall abnormalities, narrowed airways resulting from tracheobronchial defects and hypoplastic lungs [54]. We confirmed that *KCNJ2* and *SOX9* transcripts were present in human lung tissue and peripheral cells. Using publicly available microarray data [29], we established that *SOX9* is expressed in human airway epithelial cells and its expression is significantly down-regulated in smokers relative to non-smoking adults (Figure S2B). Taken together, these results suggest that *SOX9* may be involved in cigarette smoke-induced airflow obstruction, but further investigation is required to elucidate putative mechanisms.

Most of the previously implicated SNPs had genome-wide significant (or nearly significant) associations with pulmonary function in the JMA, but some were associated with pulmonary function at *P* values that did not approach the genome-wide statistical significance threshold in the JMA analysis. This pattern has two possible explanations. First, the identification of these SNPs at genome-wide statistical significance in our most recent analysis [10] required a sample size of nearly 95,000 individuals, which was obtained by combining discovery and replication cohorts, including additional genotyping on thousands of participants from studies without GWAS data. In the current analysis, the sample size is greatly reduced because of the need for detailed quantitative smoking data and because we were unable to perform additional genotyping in studies without GWAS data. Second, Manning et al.[15] showed that a meta-analysis of main SNP effects has slightly greater power than the JMA under the scenario of no interaction, so it is not surprising that a few of the prior SNP findings had varying levels of significance between our prior GWAS meta-analyses [8]–[10] and the current JMA study. While our sample size of over 50,000 study participants is large, and the study of Manning et al. [14] examining SNP-by-BMI interaction in relation to fasting insulin is nearly twice as large, identification of interactions is challenging from a statistical power perspective. Given the multiple testing issues in genome interaction testing, even larger sample sizes will likely be needed to identify gene-by-environment interactions with rare variants or with the modest effect sizes that we generally expect. Nonetheless, our findings exemplify the greater power achieved by using the joint methods, such as those reported by Manning et al. [15] and Kraft et al. [16], [33], to incorporate interaction with a clearly associated environmental risk factor. The novel genetic loci identified here for pulmonary function would have remained unknown using standard GWAS approaches.

## Methods

### Ethics statement

Nineteen independent studies contributed to our analyses. All study protocols were approved by the respective local Institutional Review Boards, and written informed consent for genetic studies was obtained from all participants included in our analyses.

### Cohort studies

Of the 19 studies contributing to our analyses, 18 studies came from the CHARGE [8], [55] or SpiroMeta [9] Consortium: Age, Gene, Environment, Susceptibility (AGES) – Reykjavik Study [56]; Atherosclerosis Risk in Communities (ARIC) Study [57]; British 1958 Birth Cohort (B58C) [58]; Coronary Artery Risk Development in Young Adults (CARDIA) [59], [60]; Cardiovascular Health Study (CHS) [61]; European Community Respiratory Health Survey (ECRHS) [62]; European Prospective Investigation into Cancer and Nutrition (EPIC, obese cases and population-based subsets) [63]; Framingham Heart Study (FHS) [64], [65]; Health, Aging, and Body Composition (Health ABC) Study [66]; Northern Finland Birth Cohort of 1966 (NFBC1966) [67], [68]; Multi-Ethnic Study of Atherosclerosis (MESA) [69], [70]; Rotterdam Study (RS-I, RS-II, and RS-III) [71]; Swiss Study on Air Pollution and Lung Diseases in Adults (SAPALDIA) [72]; Study of Health in Pomerania (SHIP) [73]; and TwinsUK [74]. We reached out to other population-based studies with GWAS genotyping and data available on cigarette smoking and pulmonary function, resulting in the inclusion of LifeLines [75]. Given the greater power needed to detect novel genetic loci with subtle gene-environment interaction regardless of the statistical method used [16], we chose to maximize statistical power to discover novel genetic loci by combining all available participants and to use the regression coefficients across the many different component studies as evidence for consistency. This approach was similarly taken by another large-scale GWAS consortium for discovering SNP main effects [24].

### Pulmonary function measurements and smoking information

All studies were included in our previous GWAS meta-analysis of pulmonary function or the follow-up replication analyses, wherein their pulmonary function testing protocols were described [10]. For studies with spirometry at a single visit (B58C, LifeLines, MESA, NFBC1966, SHIP, RS-I, RS-II, and RS-III), we analyzed FEV_1_/FVC and FEV_1_ measured at that visit. For studies with spirometry at more than one visit, we analyzed measurements from the baseline visit (AGES, ARIC, CARDIA, CHS, ECRHS, EPIC obese cases, EPIC population-based, Health ABC, and SAPALDIA) or the most recent examination with spirometry data (FHS and TwinsUK).

Smoking history (current-, past-, and never-smoking) was ascertained by questionnaire at the time of pulmonary function testing. Pack-years of smoking were calculated for current and past smokers by multiplying smoking amount (packs/day) and duration (years smoked). Table S11 presents the specific questions used to ascertain smoking history and pack-years in each of the 19 studies.

### Genotyping, quality control, and imputation

Study participants were genotyped on various genotyping platforms, and standard quality control filters for call rate, Hardy-Weinberg equilibrium p-value, MAF, and other measures were applied to the genotyped SNPs (Table S12). To generate a common set of SNPs for meta-analysis, imputation was conducted with reference haplotype panels from HapMap phase II subjects of European ancestry (CEU) (Table S12) [76]. Imputed genotype dosage values (estimated reference allele count with a fractional value ranging from 0 to 2.0) were generated for approximately 2.5 million autosomal SNPs. Among participants with genome-wide SNP genotyping data, exclusions were made due to standard quality control metrics (call rate, discordance with prior genotyping, and genotypic and phenotypic sex mismatch among others), missing pulmonary function data, or missing covariate data (Table S13).

### Statistical analysis

Our analyses included 50,047 participants from 19 studies who passed their study-specific quality control and had complete data on pulmonary function and smoking. Each study transformed the pulmonary function measures to residuals using linear regression of FEV_1_/FVC (%) and FEV_1_ (mL) on age, age^2^, sex, and standing height as predictors. Principal component eigenvectors and recruitment site were also included as covariates to adjust for population stratification (if applicable). The residuals were converted to z scores (henceforth referred to as standardized residuals). We confirmed that smoking was inversely associated with the FEV_1_/FVC and FEV_1_ standardized residuals in all 19 studies (meta-analysis β = −0.0030 and corresponding *P*<1×10^−6^ for pack-years of smoking).

The FEV_1_/FVC and FEV_1_ standardized residuals were used as the phenotypes for genome-wide association testing with linear regression models, which included the following predictor variables: imputed SNP genotype dosages, smoking history (dichotomous variable, 0 = never-smokers and 1 = ever-smokers), smoking status (dichotomous variable, 0 = never- and past-smokers and 1 = current-smokers), pack-years of smoking (continuous variable), and a SNP-by-smoking interaction product term. Two of the 19 studies (FHS and TwinsUK) had much relatedness among participants, and we took appropriate account of relatedness in the association testing (Table S12). Four regression models with interaction terms for ever-smoking or pack-years were specified in relation to standardized residuals for FEV_1_/FVC or FEV_1._ As it has long been advised in studying interactions, the regression models were designed to fully saturate the main smoking effect on pulmonary function, so that the interaction terms do not capture residual main effects [77]. In each of the 19 studies, the genome-wide analyses were implemented with robust variance estimation using the software packages indicated in Table S12.

Our analyses were aimed at finding novel loci associated with pulmonary function when considering an interaction with cigarette smoking, so we chose to implement JMA of SNP main and interactive SNP-by-smoking effects (two d.f. test of the null hypothesis β_SNP_ = 0 and β_INT_ = 0) [15]. Manning et al. previously compared the joint methods, such as JMA, with other methods that incorporate gene-environment interaction (such as screening by main effects [78] or conducting a 1 d.f. meta-analysis of the interaction product term), and they found that the joint methods offer optimal statistical power over a range of scenarios for SNP main and interactive effects [15], [33]. Therefore, our analyses centered on the JMA method, which simultaneously estimates regression coefficients for the SNP and SNP-by-smoking interaction terms, while accounting for their covariance, to generate a joint test of significance [15]. It also accounts for the unequal variances from studies of different sample sizes. Secondarily, we implemented meta-analyses of just the β coefficient from the interaction term for comparison with the JMA results. Of note, the two-step gene-environment interaction study designs by Murcray et al. [79], [80] and Gauderman et al. [81] are applicable to case-control or case-parent trio studies, respectively, and were thus not considered for our population-based studies of continuous traits.

The JMA was conducted with fixed effects on approximately 2.5 million SNPs using METAL software (version 2010-02-08) [82] and patch source code provided by Manning et al. [82]. Genomic control correction was applied by computing λ_gc_ as the ratio of the observed and expected (2 d.f.) median chi-square statistics and dividing the observed chi-square statistics by λ_gc_. SNPs having *P*_JMA_<5×10^−8^ (the standard Bonferroni-adjusted *P* value) were considered statistically significant [83]. Further correction for the four different (albeit related) JMA models yielded a conservative *P*_JMA_ threshold of 1.25×10^−8^. In addition to reporting the *P*_JMA_ for the most significant SNP from each novel locus, we used the β and standard error (SE) estimates from the JMA results to calculate the *P* values corresponding to the SNP main association (*P*_SNP_) and the SNP-by-ever-smoking interaction (*P*_INT_) [15].

### Bioinformatics analysis

Gene annotation was performed using the gene prediction tracks “UCSC Genes” and “RefSeq Genes” in the UCSC browser (http://genome.ucsc.edu). The “sno/miRNA” track from the USCS browser was used to search for any microRNA within 100 kb upstream or downstream of each SNP, and the “TFBS Conserved” track was used to search for conserved transcription factor binding sites (TFBSs) at or near the most significant SNPs. The SNAP program [84] was used to infer LD patterns, based on the HapMap phase II CEU population.

### Expression analyses

We used separate types of expression analyses to confirm the biologic plausibility of our findings. First, we carried out mRNA expression profiling to show whether or not the implicated genes are expressed in human tissues relevant to pulmonary function. The mRNA expression profiles of implicated genes were determined using reverse transcription polymerase chain reaction (RT-PCR). RNA was sourced from lung (Ambion/ABI), human bronchial epithelial cells (Clonetics) [85], and peripheral blood mononuclear cells (3H Biomedica). RNA from human airway smooth muscle cells, cultured as previously described from tissue obtained at thoracotomy [86], was extracted using a commercially available kit (Qiagen). Ethical approval for the use of primary cells was obtained from the local ethics committees. cDNA was generated using 1 µg of RNA template using random hexamers and a SuperScript kit (Invitrogen) as directed by the manufacturer. PCR assays were designed to cross intron-exon boundaries, where possible and where splice variation was known, in order to detect all variants. The *GAPDH* gene was used as a positive control for the cDNA quality, and water was used as a negative control. Primer sequences for the genes of interest are given in Table S14. All PCR were done using Platinum Taq High Fidelity (Invitrogen) with 100 ng of cDNA template in a 25 µL reaction. Cycling conditions were as follows: 94°C for 2 minutes, 35 cycles of 94°C for 45 seconds, 55°C for 30 seconds, and 68°C for 90 seconds. Following PCR, gel bands were directly sequenced to confirm the presence of the gene's transcript.

Second, we used another publically available data repository to investigate whether any of the implicated genes showed evidence for differential expression depending on smoking history. The gene expression profiles of human airway epithelium from healthy smokers (N = 10) and nonsmokers (N = 12) were obtained from the Gene Expression Omnibus site (http://www.ncbi.nlm.nih.gov/geo/) [27], [28], based on robust multichip average processing of probe intensities from Affymetrix HG-U133 Plus 2.0 microarrays (GEO dataset number GSE4498) [29]. Mean expression levels of genes around our genome-wide significant findings from the JMA were compared between smokers versus nonsmokers. The *P* value for the difference in means between smokers and nonsmokers was calculated using the nonparametric Mann-Whitney test.

Third, our genome-wide significant SNPs from novel loci were searched against an expression quantitative trait loci (eQTL) data repository based on lymphoblastoid cell lines [32], to investigate whether any of the implicated SNP variants might influence the expression of the nearby genes. *P*<5×10^−8^ was used to designate statistically significant eQTL associations.

## Supporting Information

Figure S1 Quantile–quantile plots for the genome-wide joint meta-analysis (JMA) of SNP and SNP-by-smoking interaction in relation to pulmonary function. The plots compare the observed vs. expected *P* values for JMA testing of SNPs by (A) ever-smoking in relation to FEV_1_/FVC, (B) pack-years of smoking in relation to FEV_1_/FVC, (C) ever-smoking in relation to FEV_1_, and (D) pack-years of smoking in relation to FEV_1_. The corresponding two degree-of-freedom genomic inflation factors (λ_gc_) are shown, as calculated across all SNPs before the exclusion of previously implicated SNPs. The JMA results of all SNPs were plotted (in blue), along with the SNPs remaining after exclusion of the 27 previously implicated loci (in black).
(DOCX)

Figure S2 mRNA expression profiling in human airway epithelium from healthy smokers versus nonsmokers. Expression profiles of 10 smokers (indicated in blue) and 12 nonsmokers (indicated in red) were obtained for (A) *DNER*, (B) *SOX9*, and (C) *KCNJ2*, using microarray data from the Gene Expression Omnibus site (http://www.ncbi.nlm.nih.gov/geo/) (GSE4498). The y-axes reflect the probe intensities of each gene transcript from Affymetrix HG-U133 Plus 2.0 microarrays [29], with the horizontal bold bars indicating the average probe intensities and the smaller bars indicating standard deviation. *SOX9* was represented by two different probes on the microarray; therefore, the intensities were averaged for each sample. The *P* value was calculated using the nonparametric Mann-Whitney test.
(DOCX)

Table S1 Characteristics of study participants (total N = 50,047) at the time of pulmonary function testing.
(DOCX)

Table S2 Genomic inflation factors (λ_gc_) for study-specific results (corresponding to the 1 degree of freedom SNP-by-smoking product term) in each of the four regression models.
(DOCX)

Table S3 Regions surrounding the most significant SNP from each of 27 previously implicated loci (500 kb upstream to 500 kb downstream of each SNP). These loci were excluded when identifying novel loci from the joint meta-analysis (JMA) of SNP and SNP-by-smoking interaction. The smallest P value from the JMA (*P*_JMA_) is shown, along with the corresponding JMA model from which the result was obtained.
(DOCX)

Table S4 Study-specific results for the genome-wide significant SNP rs7594321 (coded allele: T), located in the *DNER* gene. β estimates and *P* values are shown for the SNP main association (β_SNP_ and *P*_SNP_) and interactive association (β_INT_ and *P*_INT_) by smoking (ever-smoking and pack-years) in relation to FEV_1_/FVC. The *P* values corresponding to the joint test of SNP main and interactive associations are also shown.
(DOCX)

Table S5 Study-specific results for the genome-wide significant SNP rs7764819 (coded allele: T), located between the *HLA-DQB1* and *HLA-DQA2* genes. β estimates and *P* values are shown for the SNP main association (β_SNP_ and *P*_SNP_) and interactive association (β_INT_ and *P*_INT_) by smoking (ever-smoking and pack-years) in relation to FEV_1_/FVC. The *P* values corresponding to the joint test of SNP main and interactive associations are also shown.
(DOCX)

Table S6 SNPs from each of 16 chromosomal regions with *P* values between 5×10^−8^ and 1×10^−6^ for the joint meta-analysis of SNP and SNP-by-smoking (ever-smoking or pack-years) in relation to pulmonary function (FEV_1_/FVC or FEV_1_). A hyphen (“−”) indicates *P*>1×10^−6^. For each regression model, the SNP having the smallest *P*_JMA_ from each locus is shown.
(DOCX)

Table S7 SNPs with *P*<1×10^−6^ from the 1 degree-of-freedom meta-analysis of regression coefficients corresponding to the SNP-by-smoking (ever-smoking or pack-years) interaction term in relation to FEV_1_/FVC. No SNPs exceeded the standard genome-wide significance threshold (*P*<5×10^−8^). A hyphen (“−”) indicates *P*>1×10^−6^. For each regression model, the SNP having the smallest *P*_INT_ from each locus is shown.
(DOCX)

Table S8 Study-specific results for the genome-wide significant SNP rs11654749 (coded allele: T), located between the *KCNJ2* and *SOX9* genes. β estimates and *P* values are shown for the SNP main association (β_SNP_ and *P*_SNP_) and interactive association (β_INT_ and *P*_INT_) by ever-smoking in relation to FEV_1_. The *P* values corresponding to the joint test of SNP main and interactive associations are also shown.
(DOCX)

Table S9 Look-up evaluation of main SNP associations with cigarette smoking phenotypes using data generated by the Oxford-GlaxoSmithKline Consortium (N = 41,150), for the most significant SNP from each of the three novel loci implicated at genome-wide significance in the joint meta-analysis.
(DOCX)

Table S10 mRNA expression profiling of three candidate genes in the human lung and periphery. Primer sequences are provided in Table S5. A “+” sign indicates presence of the transcript, and “−” indicates its absence. All products were sequence verified.
(DOCX)

Table S11 Questionnaire data used to ascertain cigarette smoking history (ever-smoking), amount, and duration across the 19 studies. Smoking amount and duration were used together to calculate pack-years.
(DOCX)

Table S12 Details of single nucleotide polymorphism (SNP) genotyping, quality control (QC), imputation, and statistical analysis across the 19 studies.
(DOCX)

Table S13 Study participants of European descent and quality control (QC) across the 19 studies. Participants passing QC filters and having acceptable spirometry data and complete covariate data were included in the meta-analyses.
(DOCX)

Table S14 Primers for mRNA expression profiling.
(DOCX)

Text S1 Detailed explanation of joint meta-analysis significance levels, in relation to main and interactive significance.
(DOCX)
