# Supplementary figures and images for: Genome-Wide Joint Meta-Analysis of SNP and SNP-by-Smoking Interaction Identifies Novel Loci for Pulmonary Function
Source: PLoS Genet. 2012 Dec 20;8(12):e1003098. doi: 10.1371/journal.pgen.1003098 (PMC3527213; doi:10.1371/journal.pgen.1003098)

A.


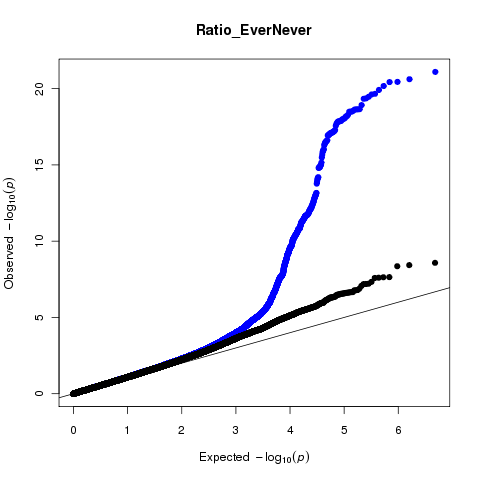


λgc=1.059

B.


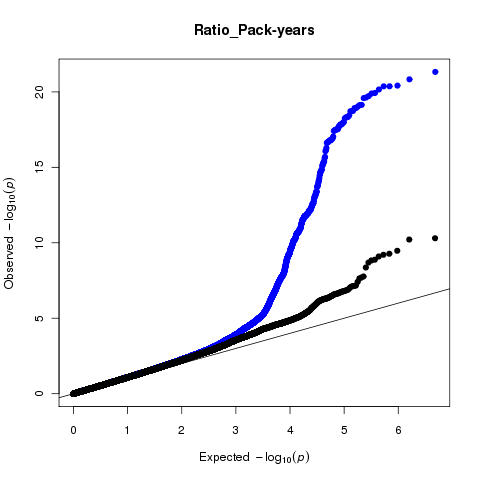


λgc=1.064

C.


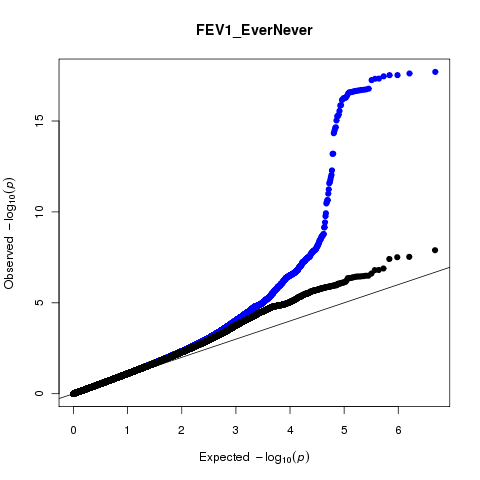


λgc=1.056

D.


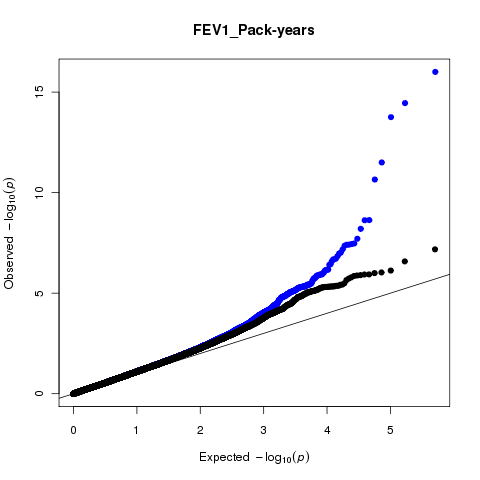


λgc=1.062

Supplement: Figure S1 — Quantile–quantile plots for the genome-wide joint meta-analysis (JMA) of SNP and SNP-by-smoking interaction in relation to pulmonary function. The plots compare the observed vs. expected P values for JMA testing of SNPs by (A) ever-smoking in relation to FEV1/FVC, (B) pack-years of smoking in relation to FEV1/FVC, (C) ever-smoking in relation to FEV1, and (D) pack-years of smoking in relation to FEV1. The corresponding two degree-of-freedom genomic inflation factors (λgc) are shown, as calculated across all SNPs before the exclusion of previously implicated SNPs. The JMA results of all SNPs were plotted (in blue), along with the SNPs remaining after exclusion of the 27 previously implicated loci (in black). (DOCX) [file pgen.1003098.s001.docx]
