# Supplementary material for: Genome-Wide Joint Meta-Analysis of SNP and SNP-by-Smoking Interaction Identifies Novel Loci for Pulmonary Function
Source: PLoS Genet. 2012 Dec 20;8(12):e1003098. doi: 10.1371/journal.pgen.1003098 (PMC3527213; doi:10.1371/journal.pgen.1003098)
Supplement: Figure S2 — mRNA expression profiling in human airway epithelium from healthy smokers versus nonsmokers. Expression profiles of 10 smokers (indicated in blue) and 12 nonsmokers (indicated in red) were obtained for (A) DNER, (B) SOX9, and (C) KCNJ2, using microarray data from the Gene Expression Omnibus site (http://www.ncbi.nlm.nih.gov/geo/) (GSE4498). The y-axes reflect the probe intensities of each gene transcript from Affymetrix HG-U133 Plus 2.0 microarrays [29], with the horizontal bold bars indicating the average probe intensities and the smaller bars indicating standard deviation. SOX9 was represented by two different probes on the microarray; therefore, the intensities were averaged for each sample. The P value was calculated using the nonparametric Mann-Whitney test. (DOCX) [file pgen.1003098.s002.docx]

A.

B.

C.
