# Supplementary material for: Genome-Wide Joint Meta-Analysis of SNP and SNP-by-Smoking Interaction Identifies Novel Loci for Pulmonary Function
Source: PLoS Genet. 2012 Dec 20;8(12):e1003098. doi: 10.1371/journal.pgen.1003098 (PMC3527213; doi:10.1371/journal.pgen.1003098)
Supplement: Table S1 — Characteristics of study participants (total N = 50,047) at the time of pulmonary function testing. (DOCX) [file pgen.1003098.s003.docx]

| **Study (Country of origin)** | **Years of spirometry data collection** | **N, total** | **N, males (%)** | **N, females (%)** | **Age (years) [mean (SD)]** | **Height (cm) [mean (SD)]** | **N, never-smokers (%)** | **N,**  **ever-smokers (%)** | **Pack-years among ever-smokers**  **[mean (SD)]** | **FEV_1_ (mL) [mean (SD)]** | **FVC (mL) [mean (SD)]** | **FEV_1_/FVC (%)**  **[mean (SD)]** |
| --- | --- | --- | --- | --- | --- | --- | --- | --- | --- | --- | --- | --- |
| AGES (Iceland)^1^ | 2002-2004 | 1,696 | 689 (40.6) | 1,007 (59.4) | 76.2 (5.6) | 166.7 (9.4) | 813 (47.9) | 883 (52.1) | 24.5 (21.9) | 2,128 (690) | 2,865 (848) | 73.9 (10.5) |
| ARIC  (US)^1^ | 1987-1989 | 8,934 | 4,225  (47.3) | 4,709 (52.7) | 54.3 (5.7) | 168.8 (9.4) | 3,620 (40.5) | 5,314 (59.5) | 28.9 (21.6) | 2,943 (744) | 3,993 (980) | 73.7 (7.9) |
| B58C (UK)^1^ | 2002-2004 | 4,605 | 2,291  (49.8) | 2,314  (50.3) | 44.5  (0.4) | 169.2  (9.3) | 1,376  (29.9) | 3,229  (70.1) | 15.7 (12.1) | 3,288  (757) | 4,164  (980) | 79.5  (8.1) |
| CARDIA (US)^2^ | 1985-1987 | 1,605 | 758 (47.2) | 847 (52.8) | 25.6 (3.3) | 171.3 (9.3) | 932 (58.1) | 673 (41.9) | 5.5 (5.5) | 3,684 (810) | 4,702 (1,010) | 82.2  (6.4) |
| CHS  (US)^1^ | 1989-1990 | 3,140 | 1226 (39.0) | 1914 (61.0) | 72.3 (5.4) | 164.6 (9.4) | 1543 (49.1) | 1597 (50.9) | 33.2 (26.9) | 2,116 (659) | 3,005 (866) | 70.5 (10.5) |
| ECRHS (European countries)^1,3^ | 1992-1993 | 1,573 | 784 (49.2) | 810 (50.8) | 33.9 (7.2) | 170.7 (9.5) | 699 (43.9) | 895 (56.1) | 12.8 (12.6) | 3,778 (825) | 4,595 (1029) | 82.6 (6.6) |
| EPIC obese cases  (European countries)^1,4^ | 1993-1997 | 1,084 | 458  (42.3) | 626 (57.8) | 59.1 (8.8) | 165.93 (9.24) | 489 (44.3) | 595 (54.9) | 18.2 (14.1) | 2,355 (694) | 2,839 (872) | 83.83 (10.2) |
| EPIC population-based  (European countries)^1,4^ | 1993-1997 | 2,294 | 1,064 (46.4) | 1,230 (53.6) | 59.1 (9.0) | 167.0 (8.9) | 1,062 (46.3) | 1,232 (53.7) | 15.8 (13.4) | 2,500 (718) | 3,042 (903) | 83.1 (10.8) |
| FHS  (US)^1^ | Cohort: 1974-, Offspring: 1984-, Gen3: 2002- | 7,694 | 3,544 (46.1) | 4,150 (53.9) | 51.9 (14.6) | 168.5 (9.7) | 3,556 (46.2) | 4,138 (53.8) | 22.8 (21.5) | 3,038 (944) | 4,025 (1,144) | 75.1 (8.0) |
| Health ABC (US)^1^ | 1997-1998 | 1,472 | 786 (53.4) | 686 (46.6) | 73.7 (2.8) | 167.1 (9.3) | 641 (43.6) | 831 (56.5) | 36.6 (32.0) | 2,312 (656) | 3,113 (812) | 74.1 (7.7) |
| LifeLines (The Netherlands)^2^ | 2006-2009 | 2,616 | 1,048 (40.1) | 1,568 (59.9) | 54.2 (9.5) | 173.0 (9.1) | 981 (37.7) | 1,621 (52.3) | 14.5 (12.6) | 3,172 (804) | 4,233 (1,007) | 75.0 (7.5) |
| MESA  (US)^2^ | 2004-2006 | 1,403 | 688  (49.0) | 715  (51.0) | 66.0  (9.7) | 168.5  (9.7) | 636  (45.3) | 767  (54.7) | 27.5  (24.4) | 2,566  (763) | 3,505  (999.6) | 73.4  (8.4) |
| NFBC1966 (Finland)^1^ | 1997 | 3,564 | 1,764 (49.5) | 1,800 (50.5) | 31 (0) | 171.5 (9.3) | 1,648 (46.2) | 1916 (53.8) | 9.6 (7.9) | 3,969 (791) | 4,744 (989) | 84.1 (6.5) |
| RS-I  (The Netherlands)^1^ | 2002-2004 | 1,196 | 491 (41.4) | 705  (58.9) | 74.4  (5.7) | 166.7  (8.9) | 408  (34.1) | 788  (65.9) | 24.9 (19.6) | 2,334  (735) | 3,183  (927) | 73.2 (8.2) |
| RS-II  (The Netherlands)^1^ | 2004-2005 | 840 | 373  (44.4) | 467  (55.6) | 67.1  (6.2) | 168.3  (8.9) | 287 (34.2) | 553  (65.8) | 23.1 (19.2) | 2,716  (779) | 3,615  (1,077) | 75.9 (9.1) |
| RS-III  (The Netherlands)^2^ | 2006-2008 | 1,224 | 529  (43.2) | 695 (56.8) | 56.6  (5.6) | 171.2  (9.3) | 425 (34.7) | 799 (65.3) | 18.2 (16.0) | 3,159  (851) | 4,059  (1,138) | 78.4 (9.0) |
| SAPALDIA (Switzerland)^2^ | 1991 | 1,333 | 632  (47.4) | 701  (52.6) | 41.1 (11.2) | 169.4 (9.0) | 626 (47.0) | 707 (53.0) | 17.3 (18.0) | 3,524 (860) | 4,494 (1,038) | 78.5 (8.2) |
| SHIP (Germany)^1^ | 2002-2006 | 1,768 | 863 (48.8) | 905 (51.2) | 52.4 (13.6) | 169.7 (9.1) | 770 (43.6) | 998 (56.4) | 12.8 (12.0) | 3,280 (894) | 3,869 (1,030) | 84.8 (6.5) |
| TwinsUK (UK)^1^ | 1995-2010 | 2,006 | 0 | 2,006 (100) | 54.2 (14.1) | 161.8 (6.4) | 1,242 (61.9) | 764 (38.1) | 13.7 (21.4) | 2,599 (606) | 3,251 (650) | 79.7 (7.7) |

AGES, Age, Gene/Environment Susceptibility; ARIC, Atherosclerosis Risk in Communities; B58C, British 1958 Cohort; CARDIA, Coronary Artery Risk Development in Young Adults; CHS, Cardiovascular Health Study; ECRHS, European Community Respiratory Health Survey; EPIC, European Prospective Investigation into Cancer and Nutrition; FEV_1_, forced expiratory volume in the first second; FVC, forced vital capacity; FHS, Framingham Heart Study; Health ABC, Health, Aging, and Body Composition Study; MESA, Multi-Ethnic Study of Atherosclerosis; NFBC1966, Northern Finland Birth Cohort of 1966; RS, Rotterdam Study (cohorts I-III); SAPALDIA, Swiss Study on Air Pollution and Lung Diseases in Adults; SD, standard deviation; SHIP, Study of Health in Pomerania; SNP, single nucleotide polymorphism.

^1^ Study included in the previous GWAS meta-analysis of FEV_1_/FVC and FEV_1_ [[1](#_ENREF_1)].

^2^ Replication study for the previous GWAS meta-analysis of FEV_1_/FVC and FEV_1_ [[1](#_ENREF_1)].

^3^ The genetics data used in ECRHS includes participants from 16 centers across 8 European countries (Estonia, France, Germany, Norway, Spain, Sweden, Switzerland, and UK).

^4^ EPIC includes participants from 10 European countries: Denmark, France, Germany, Greece, Italy, The Netherlands, Norway, Spain, Sweden, and the United Kingdom.

**Reference**

1. Soler Artigas M, Loth DW, Wain LV, Gharib SA, Obeidat M, et al. (2011) Genome-wide association and large-scale follow up identifies 16 new loci influencing lung function. Nat Genet 43: 1082-1090.
