# Supplementary material for: Genome-Wide Joint Meta-Analysis of SNP and SNP-by-Smoking Interaction Identifies Novel Loci for Pulmonary Function
Source: PLoS Genet. 2012 Dec 20;8(12):e1003098. doi: 10.1371/journal.pgen.1003098 (PMC3527213; doi:10.1371/journal.pgen.1003098)
Supplement: Table S2 — Genomic inflation factors (λgc) for study-specific results (corresponding to the 1 degree of freedom SNP-by-smoking product term) in each of the four regression models. (DOCX) [file pgen.1003098.s004.docx]

| **Study** | **FEV_1_/FVC** | | **FEV_1_** | |
| --- | --- | --- | --- | --- |
|  | **Ever-Smoking** | **Pack-Years of Smoking** | **Ever-Smoking** | **Pack-Years of Smoking** |
| AGES | 1.03 | 1.07 | 1.02 | 1.09 |
| ARIC | 1.00 | 1.02 | 1.00 | 1.02 |
| B58C | 1.02 | 1.03 | 1.02 | 1.01 |
| CARDIA | 1.01 | 1.04 | 1.01 | 1.04 |
| CHS | 1.01 | 1.04 | 1.02 | 1.06 |
| ECRHS | 1.01 | 1.03 | 1.04 | 1.06 |
| EPIC obese cases | 1.02 | 1.05 | 1.04 | 1.05 |
| EPIC population-based | 1.01 | 1.03 | 1.01 | 1.02 |
| FHS | 1.01 | 1.03 | 1.03 | 1.04 |
| Health ABC | 1.00 | 1.03 | 1.00 | 1.04 |
| LifeLines | 0.99 | 1.00 | 1.01 | 1.03 |
| MESA | 0.99 | 1.02 | 1.03 | 1.04 |
| NFBC1966 | 1.00 | 1.02 | 1.02 | 1.02 |
| RS-I | 1.04 | 1.02 | 1.01 | 1.03 |
| RS-II | 1.03 | 1.06 | 1.03 | 1.08 |
| RS-III | 1.03 | 1.05 | 1.02 | 1.04 |
| SAPALDIA | 1.01 | 1.03 | 1.02 | 1.06 |
| SHIP | 1.02 | 1.04 | 0.99 | 1.03 |
| TwinsUK | 1.02 | 1.06 | 1.01 | 1.06 |

AGES, Age, Gene/Environment Susceptibility; ARIC, Atherosclerosis Risk in Communities; B58C, British 1958 Cohort; CARDIA, Coronary Artery Risk Development in Young Adults; CHS, Cardiovascular Health Study; ECRHS, European Community Respiratory Health Survey; EPIC, European Prospective Investigation into Cancer and Nutrition; FEV_1_, forced expiratory volume in the first second; FVC, forced vital capacity; FHS, Framingham Heart Study; Health ABC, Health, Aging, and Body Composition Study; MESA, Multi-Ethnic Study of Atherosclerosis; NFBC1966, Northern Finland Birth Cohort of 1966; RS, Rotterdam Study (cohorts I-III); SAPALDIA, Swiss Study on Air Pollution and Lung Diseases in Adults; SHIP, Study of Health in Pomerania; SNP, single nucleotide polymorphism.
