# Supplementary material for: Genome-Wide Joint Meta-Analysis of SNP and SNP-by-Smoking Interaction Identifies Novel Loci for Pulmonary Function
Source: PLoS Genet. 2012 Dec 20;8(12):e1003098. doi: 10.1371/journal.pgen.1003098 (PMC3527213; doi:10.1371/journal.pgen.1003098)
Supplement: Table S3 — Regions surrounding the most significant SNP from each of 27 previously implicated loci (500 kb upstream to 500 kb downstream of each SNP). These loci were excluded when identifying novel loci from the joint meta-analysis (JMA) of SNP and SNP-by-smoking interaction. The smallest P value from the JMA (P JMA) is shown, along with the corresponding JMA model from which the result was obtained. (DOCX) [file pgen.1003098.s005.docx]

| **SNP**^1^ | **Chr** | **Base pair position (NCBI build 36)** | **Gene / nearby gene(s)** | **Original Reference** | **Smallest *P*_JMA_** | **Model with the smallest *P*_JMA_** | |
| --- | --- | --- | --- | --- | --- | --- | --- |
|  |  |  |  |  |  | **Pulmonary function measure** | **Smoking metric** |
| rs2284746 | 1 | 17,179,262 | *MFAP2* | [[1](#_ENREF_1)] | 2.14x10^-11^ | FEV_1_/FVC | Pack-years |
| rs993925 | 1 | 216,926,691 | *TGFβ2* | [[1](#_ENREF_1)] | 1.55x10^-3^ | FEV_1_ | Ever-smoking |
| rs2571445 | 2 | 218,391,399 | *TNS1* | [[2](#_ENREF_2)] | 7.81x10^-9^ | FEV_1_ | Ever-smoking |
| rs1435867 | 2 | 229,219,173 | *PID1* | [[3](#_ENREF_3)] | 2.02x10^-9^ | FEV_1_/FVC | Ever-smoking |
| rs12477314 | 2 | 239,542,085 | *HDAC4* | [[1](#_ENREF_1)] | 1.81x10^-7^ | FEV_1_/FVC | Pack-years |
| rs1529672 | 3 | 25,495,586 | *RARB* | [[1](#_ENREF_1)] | 6.94x10^-11^ | FEV_1_/FVC | Pack-years |
| rs1344555 | 3 | 170,782,913 | *MECOM* | [[1](#_ENREF_1)] | 3.75x10^-6^ | FEV_1_ | Pack-years |
| rs2869967 | 4 | 90,088,355 | *FAM13A* | [[3](#_ENREF_3)] | 5.48x10^-11^ | FEV_1_/FVC | Ever-smoking |
| rs17331332 | 4 | 107,027,556 | *INTS12/ GSTCD/ NPNT* | [[2](#_ENREF_2),[3](#_ENREF_3)] | 1.34x10^-16^ | FEV_1_ | Ever-smoking |
| rs1980057 | 4 | 145,705,188 | *HHIP* | [[2](#_ENREF_2),[3](#_ENREF_3)] | 5.11x10^-18^ | FEV_1_/FVC | Ever-smoking |
| rs153916 | 5 | 95,062,456 | *SPATA9* | [[1](#_ENREF_1)] | 6.14x10^-4^ | FEV_1_/FVC | Ever-smoking |
| rs11168048 | 5 | 147,822,546 | *HTR4* | [[2](#_ENREF_2),[3](#_ENREF_3)] | 5.13x10^-17^ | FEV_1_/FVC | Ever-smoking |
| rs2277027 | 5 | 156,864,954 | *ADAM19* | [[3](#_ENREF_3)] | 1.54x10^-11^ | FEV_1_/FVC | Ever-smoking |
| rs6903823 | 6 | 28,430,275 | *ZSCAN3 / ZNF323* | [[1](#_ENREF_1)] | 3.71x10^-4^ | FEV_1_ | Pack-years |
| rs2857595 | 6 | 31,676,448 | *NCR3* | [[1](#_ENREF_1)] | 7.74x10^-8^ | FEV_1_/FVC | Ever-smoking |
| rs2070600 | 6 | 32,259,421 | *AGER* | [[2](#_ENREF_2),[3](#_ENREF_3)] | 1.47x10^-21^ | FEV_1_/FVC | Pack-years |
| rs2798641 | 6 | 109,374,743 | *ARMC2* | [[1](#_ENREF_1)] | 1.81x10^-7^ | FEV_1_ | Pack-years |
| rs3817928 | 6 | 142,792,209 | *GPR126* | [[3](#_ENREF_3)] | 2.75x10^-12^ | FEV_1_/FVC | Pack-years |
| rs16909898 | 9 | 97,270,829 | *PTCH1* | [[3](#_ENREF_3)] | 8.20x10^-12^ | FEV_1_/FVC | Ever-smoking |
| rs7068966 | 10 | 12,317,998 | *CDC123* | [[1](#_ENREF_1)] | 1.50x10^-11^ | FEV_1_/FVC | Ever-smoking |
| rs11001819 | 10 | 77,985,230 | *C10orf11* | [[1](#_ENREF_1)] | 5.38x10^-8^ | FEV_1_ | Ever-smoking |
| rs11172113 | 12 | 55,813,550 | *LRP1* | [[1](#_ENREF_1)] | 8.27x10^-6^ | FEV_1_/FVC | Pack-years |
| rs1036429 | 12 | 94,795,559 | *CCDC38* | [[1](#_ENREF_1)] | 1.47x10^-7^ | FEV_1_/FVC | Ever-smoking |
| rs12899618 | 15 | 69,432,174 | *THSD4* | [[2](#_ENREF_2)] | 4.30x10^-21^ | FEV_1_/FVC | Pack-years |
| rs12447804 | 16 | 56,632,783 | *MMP15* | [[1](#_ENREF_1)] | 3.84x10^-8^ | FEV_1_/FVC | Ever-smoking |
| rs2865531 | 16 | 73,947,817 | *CFDP1* | [[1](#_ENREF_1)] | 1.94x10^-8^ | FEV_1_/FVC | Pack-years |
| rs9978142 | 21 | 34,574,109 | *KCNE2* | [[1](#_ENREF_1)] | 3.17x10^-5^ | FEV_1_/FVC | Ever-smoking |

JMA, joint meta-analysis; SNP, single nucleotide polymorphism. ^1^SNPs are sorted by chromosomal position.

**References**

1. Soler Artigas M, Loth DW, Wain LV, Gharib SA, Obeidat M, et al. (2011) Genome-wide association and large-scale follow up identifies 16 new loci influencing lung function. Nat Genet 43: 1082-1090.

2. Repapi E, Sayers I, Wain LV, Burton PR, Johnson T, et al. (2010) Genome-wide association study identifies five loci associated with lung function. Nat Genet 42: 36-44.

3. Hancock DB, Eijgelsheim M, Wilk JB, Gharib SA, Loehr LR, et al. (2010) Meta-analyses of genome-wide association studies identify multiple loci associated with pulmonary function. Nat Genet 42: 45-52.
