# Supplementary material for: Genome-Wide Joint Meta-Analysis of SNP and SNP-by-Smoking Interaction Identifies Novel Loci for Pulmonary Function
Source: PLoS Genet. 2012 Dec 20;8(12):e1003098. doi: 10.1371/journal.pgen.1003098 (PMC3527213; doi:10.1371/journal.pgen.1003098)
Supplement: Table S5 — Study-specific results for the genome-wide significant SNP rs7764819 (coded allele: T), located between the HLA-DQB1 and HLA-DQA2 genes. β estimates and P values are shown for the SNP main association (βSNP and P SNP) and interactive association (βINT and P INT) by smoking (ever-smoking and pack-years) in relation to FEV1/FVC. The P values corresponding to the joint test of SNP main and interactive associations are also shown. (DOCX) [file pgen.1003098.s007.docx]

| **Study** | **N** | **Coded allele frequency** | **Imputation quality metric** | **Ever-smoking** | | | | | **Pack-years** | | | | |
| --- | --- | --- | --- | --- | --- | --- | --- | --- | --- | --- | --- | --- | --- |
|  |  |  |  | **β_SNP_** | ***P*_SNP_** | **β_INT_** | ***P*_INT_** | **Joint *P*^1^** | **β_SNP_** | ***P*_SNP_** | **β_INT_** | ***P*_INT_** | **Joint *P*^1^** |
| AGES | 1,696 | 0.87 | 0.97 | 0.0041 | 0.95 | -0.038 | 0.70 | 0.88 | 0.0091 | 0.87 | -0.0050 | 0.42 | 0.68 |
| ARIC | 8,934 | 0.89 | 0.98 | -0.028 | 0.40 | -0.025 | 0.58 | 0.18 | -0.029 | 0.30 | -0.0007 | 0.52 | 0.17 |
| B58C | 4,605 | 0.88 | 1.00 | -0.049 | 0.31 | -0.026 | 0.68 | 0.10 | -0.052 | 0.17 | -0.000063 | 0.61 | 0.097 |
| CARDIA | 1,605 | 0.90 | 0.99 | -0.073 | 0.32 | -0.027 | 0.82 | 0.36 | -0.052 | 0.42 | -0.015 | 0.41 | 0.33 |
| CHS | 3,140 | 0.88 | 0.43 | -0.044 | 0.58 | -0.0020 | 0.99 | 0.73 | 0.0015 | 0.98 | -0.0026 | 0.26 | 0.42 |
| ECHRS | 1,594 | 0.90 | 0.98 | 0.027 | 0.76 | -0.20 | 0.086 | 0.074 | -0.050 | 0.47 | -0.0036 | 0.48 | 0.31 |
| EPIC obese cases | 1,084 | 0.89 | 0.84 | -0.085 | 0.41 | -0.039 | 0.78 | 0.33 | -0.15 | 0.087 | 0.0039 | 0.50 | 0.22 |
| EPIC population-based | 2,294 | 0.89 | 0.84 | -0.058 | 0.39 | 0.014 | 0.89 | 0.58 | -0.028 | 0.63 | -0.0031 | 0.50 | 0.49 |
| FHS | 7,694 | 0.90 | 0.86 | -0.096 | 0.017 | 0.040 | 0.46 | 0.033 | -0.073 | 0.026 | -0.00013 | 0.95 | 0.044 |
| Health ABC | 1,472 | 0.90 | 1.00 | 0.030 | 0.72 | -0.17 | 0.14 | 0.21 | -0.020 | 0.78 | -0.0020 | 0.30 | 0.34 |
| LifeLines | 2,616 | NA | NA | NA | NA | NA | NA | NA | NA | NA | NA | NA | NA |
| MESA | 1,403 | 0.91 | 0.90 | -0.25 | 0.0057 | 0.076 | 0.54 | 0.0027 | -0.14 | 0.059 | -0.0041 | 0.16 | 0.0033 |
| NFBC1966 | 3,564 | 0.83 | 0.94 | -0.050 | 0.29 | -0.057 | 0.38 | 0.034 | -0.068 | 0.080 | -0.0021 | 0.59 | 0.040 |
| RS-I | 1,196 | 0.90 | 1.00 | -0.13 | 0.14 | 0.035 | 0.76 | 0.17 | -0.16 | 0.029 | 0.0030 | 0.33 | 0.084 |
| RS-II | 840 | 0.90 | 1.00 | -0.20 | 0.12 | 0.23 | 0.16 | 0.29 | -0.055 | 0.59 | -0.000038 | 0.99 | 0.78 |
| RS-III | 1,224 | 0.91 | 1.00 | -0.15 | 0.20 | 0.057 | 0.69 | 0.24 | -0.17 | 0.028 | 0.0053 | 0.35 | 0.081 |
| SAPALDIA | 1,333 | 0.90 | 0.98 | -0.062 | 0.48 | 0.12 | 0.33 | 0.62 | -0.0082 | 0.91 | 0.0010 | 0.79 | 0.97 |
| SHIP | 1,768 | 0.91 | 0.94 | -0.090 | 0.33 | 0.0059 | 0.96 | 0.36 | -0.049 | 0.49 | -0.0054 | 0.34 | 0.23 |
| TwinsUK | 2,006 | 0.88 | 0.92 | -0.068 | 0.25 | 0.052 | 0.60 | 0.51 | -0.070 | 0.19 | 0.0028 | 0.53 | 0.42 |

AGES, Age, Gene/Environment Susceptibility; ARIC, Atherosclerosis Risk in Communities; B58C, British 1958 Cohort; CARDIA, Coronary Artery Risk Development in Young Adults; CHS, Cardiovascular Health Study; ECRHS, European Community Respiratory Health Survey; EPIC, European Prospective Investigation into Cancer and Nutrition; FEV_1_, forced expiratory volume in the first second; FVC, forced vital capacity; FHS, Framingham Heart Study; Health ABC, Health, Aging, and Body Composition Study; INT, interaction; MESA, Multi-Ethnic Study of Atherosclerosis; NA, not available; NFBC1966, Northern Finland Birth Cohort of 1966; RS, Rotterdam Study (cohorts I-III); SAPALDIA, Swiss Study on Air Pollution and Lung Diseases in Adults; SHIP, Study of Health in Pomerania; SNP, single nucleotide polymorphism.

^1^The joint *P* value corresponds to the 2 degrees-of-freedom joint test of the SNP main and interactive effect by Kraft et al. [[1](#_ENREF_1)]. The comparable 2 degrees-of-freedom joint test by Manning et al. [[2](#_ENREF_2)] is only applicable in the meta-analysis setting, as presented in the manuscript when combining results from all 19 studies.

**References**

1. Kraft P, Yen YC, Stram DO, Morrison J, Gauderman WJ (2007) Exploiting gene-environment interaction to detect genetic associations. Hum Hered 63: 111-119.

2. Manning AK, LaValley M, Liu C-T, Rice K, An P, et al. (2011) Meta-analysis of gene-environment interaction: joint estimation of SNP and SNPxEnvironment regression coefficients. Genet Epidemiol 35: 11-18.
