# Supplementary material for: Genome-Wide Joint Meta-Analysis of SNP and SNP-by-Smoking Interaction Identifies Novel Loci for Pulmonary Function
Source: PLoS Genet. 2012 Dec 20;8(12):e1003098. doi: 10.1371/journal.pgen.1003098 (PMC3527213; doi:10.1371/journal.pgen.1003098)
Supplement: Table S6 — SNPs from each of 16 chromosomal regions with P values between 5×10^−8^ and 1×10^−6^ for the joint meta-analysis of SNP and SNP-by-smoking (ever-smoking or pack-years) in relation to pulmonary function (FEV1/FVC or FEV1). A hyphen (“−”) indicates P>1×10^−6^. For each regression model, the SNP having the smallest P JMA from each locus is shown. (DOCX) [file pgen.1003098.s008.docx]

| **SNP**  **(coded allele)** | **Chr** | **Base pair position** | **SNP type** | **Gene / closest gene(s)** | ***P_JMA_* across four interaction models of smoking in relation to pulmonary function** | | | |
| --- | --- | --- | --- | --- | --- | --- | --- | --- |
|  |  |  |  |  | **FEV_1_/FVC** | | **FEV_1_** | |
|  |  |  |  |  | **Ever-Smoking** | **Pack-years** | **Ever-Smoking** | **Pack-years** |
| rs8056446 (A) | 16 | 76,745,997 | intronic | *WWOX* | - | - | - | 7.64x10^-8^ |
| rs8040868 (T) | 15 | 76,698,236 | synonymous | *CHRNA3* | - | 9.03x10^-8^ | - | 7.29x10^-7^ |
| rs12716850 (A) | 16 | 76,745,404 | intronic | *WWOX* | - | - | 1.30x10^-7^ | - |
| rs9368649 (A) | 6 | 31,046,862 | intergenic | *DPCR1 / MUC21* | 1.30x10^-7^ | 1.32x10^-7^ | - | - |
| rs1928168 (T) | 6 | 22,125,717 | intergenic | *SOX4 / PRL* | 1.65x10^-7^ | - | - | - |
| rs2078543 (A) | 6 | 22,104,839 | intergenic | *SOX4 / PRL* | - | 1.86x10^-7^ | - | - |
| rs2544527 (T) | 2 | 15,843,619 | intergenic | *DDX1 / MYCN* | - | 2.12x10^-7^ | - | - |
| rs2456203 (T) | 5 | 52,215,547 | intronic | *ITGA1* | - | 2.31x10^-7^ | - | - |
| rs8089099 (A) | 18 | 10,068,071 | intergenic | *TXNDC2 / VAPA* | 3.19x10^-7^ | 4.38x10^-7^ | - | - |
| rs10751226 (T) | 11 | 72,989,900 | intergenic | *FAM168A / PLEKHB* | 4.43x10^-7^ | 3.35x10^-7^ | - | - |
| rs2027760 (A) | 11 | 72,714,129 | intronic | *ARHGEF* | 3.53x10^-7^ | 9.76x10^-7^ | - | - |
| rs3003429 (T) | 1 | 17,464,266 | intronic | *PADI3* | - | - | - | 3.82x10^-7^ |
| rs3734729 (A) | 6 | 150,612,560 | 3’ untranslated | *PPP1R1* | - | - | 3.86x10^-7^ | 6.95x10^-7^ |
| rs2252711 (T) | 6 | 29,734,300 | intronic | *MOG* | 4.19x10^-7^ | - | - | - |
| rs2206030 (T) | 6 | 35,512,332 | intergenic | *PPARD / FANCE* | 4.55x10^-7^ | - | - | - |
| rs10513821 (A) | 3 | 189,026,036 | intergenic | *RTP2 / BCL6* | - | 4.93x10^-7^ | - | - |
| rs10777288 (A) | 12 | 89,997,929 | intergenic | *KERA / LUM* | 4.96x10^-7^ | - | - | - |
| rs13290997 (A) | 9 | 118,354,038 | intronic | *ASTN2* | - | - | - | 4.99x10^-7^ |

FEV_1_, forced expiratory volume in the first second; FVC, forced vital capacity; JMA, joint meta-analysis; SNP, single nucleotide polymorphism.
