# Supplementary material for: Genome-Wide Joint Meta-Analysis of SNP and SNP-by-Smoking Interaction Identifies Novel Loci for Pulmonary Function
Source: PLoS Genet. 2012 Dec 20;8(12):e1003098. doi: 10.1371/journal.pgen.1003098 (PMC3527213; doi:10.1371/journal.pgen.1003098)
Supplement: Table S7 — SNPs with P<1×10^−6^ from the 1 degree-of-freedom meta-analysis of regression coefficients corresponding to the SNP-by-smoking (ever-smoking or pack-years) interaction term in relation to FEV1/FVC. No SNPs exceeded the standard genome-wide significance threshold (P<5×10^−8^). A hyphen (“−”) indicates P>1×10^−6^. For each regression model, the SNP having the smallest P INT from each locus is shown. (DOCX) [file pgen.1003098.s009.docx]

| **SNP**  **(coded allele)** | **Chr** | **Base pair position** | **SNP type** | **Gene / closest gene(s)** | ***P*_INT_ across four interaction models of smoking in relation to pulmonary function** | | | |
| --- | --- | --- | --- | --- | --- | --- | --- | --- |
|  |  |  |  |  | **FEV_1_/FVC** | | **FEV_1_** | |
|  |  |  |  |  | **Ever-Smoking** | **Pack-years** | **Ever-Smoking** | **Pack-years** |
| rs3003429 (T) | 1 | 17,464,266 | intronic | *PADI3* | - | - | - | 1.13x10^-7^ |
| rs9862443 (T) | 3 | 153,003,691 | intergenic | *AADACL2 / AADAC* | 7.26x10^-7^ | - | - | - |
| rs7941377 (A) | 11 | 2,656,737 | intronic | *KCNQ1* | - | 6.55x10^-7^ | - | - |
| rs10147359 (T) | 14 | 34,425,496 | intergenic | *BAZ1A / C14orf19* | - | - | - | 9.30x10^-7^ |
| rs10151462 (A) | 14 | 50,837,300 | intergenic | *TXNDC1 /*  *FRMD6* | - | - | - | 2.39x10^-7^ |
| rs11905801 (A) | 20 | 61,941,677 | intergenic | *ZBTB46 / C20orf135* | - | - | 5.14x10^-7^ | - |
