# Supplementary material for: Genome-Wide Joint Meta-Analysis of SNP and SNP-by-Smoking Interaction Identifies Novel Loci for Pulmonary Function
Source: PLoS Genet. 2012 Dec 20;8(12):e1003098. doi: 10.1371/journal.pgen.1003098 (PMC3527213; doi:10.1371/journal.pgen.1003098)
Supplement: Table S8 — Study-specific results for the genome-wide significant SNP rs11654749 (coded allele: T), located between the KCNJ2 and SOX9 genes. β estimates and P values are shown for the SNP main association (βSNP and P SNP) and interactive association (βINT and P INT) by ever-smoking in relation to FEV1. The P values corresponding to the joint test of SNP main and interactive associations are also shown. (DOCX) [file pgen.1003098.s010.docx]

| **Study** | **N** | **Coded allele frequency** | **Imputation quality metric** | **β_SNP_** | ***P*_SNP_** | **β_INT_** | ***P*_INT_** | **Joint *P*^1^** |
| --- | --- | --- | --- | --- | --- | --- | --- | --- |
| AGES | 1,696 | 0.39 | 1.00 | 0.0041 | 0.94 | -0.0089 | 0.90 | 0.99 |
| ARIC | 8,934 | 0.40 | 0.99 | -0.022 | 0.29 | -0.025 | 0.37 | 0.025 |
| B58C | 4,605 | 0.41 | 0.99 | 0.048 | 0.16 | -0.070 | 0.097 | 0.25 |
| CARDIA | 1,605 | 0.37 | 1.00 | -0.077 | 0.095 | -0.015 | 0.83 | 0.050 |
| CHS | 3,140 | 0.37 | 1.01 | -0.061 | 0.066 | 0.036 | 0.47 | 0.15 |
| ECHRS | 1,594 | 0.37 | 0.97 | -0.064 | 0.25 | 0.033 | 0.66 | 0.42 |
| EPIC obese cases | 1,084 | 0.39 | 0.99 | -0.056 | 0.35 | 0.084 | 0.31 | 0.57 |
| EPIC population-based | 2,294 | 0.40 | 0.99 | -0.0015 | 0.97 | -0.097 | 0.11 | 0.053 |
| FHS | 7,694 | 0.39 | 1.01 | -0.0017 | 0.95 | -0.044 | 0.17 | 0.15 |
| Health ABC | 1,472 | 0.37 | 0.99 | 0.0026 | 0.96 | -0.015 | 0.84 | 0.97 |
| LifeLines | 2,616 | 0.56 | 0.64 | -0.066 | 0.14 | -0.020 | 0.72 | 0.019 |
| MESA | 1,403 | 0.36 | 1.00 | -0.14 | 0.0055 | 0.030 | 0.67 | 0.0020 |
| NFBC1966 | 3,564 | 0.32 | 1.00 | -0.064 | 0.080 | 0.042 | 0.42 | 0.18 |
| RS-I | 1,196 | 0.39 | 1.00 | 0.044 | 0.48 | -0.096 | 0.23 | 0.46 |
| RS-II | 840 | 0.39 | 0.99 | -0.13 | 0.062 | 0.16 | 0.092 | 0.15 |
| RS-III | 1,224 | 0.42 | 1.00 | -0.010 | 0.86 | -0.034 | 0.66 | 0.67 |
| SAPALDIA | 1,333 | 0.38 | 0.98 | -0.016 | 0.78 | 0.0072 | 0.93 | 0.95 |
| SHIP | 1,768 | 0.37 | 1.00 | -0.039 | 0.45 | -0.071 | 0.30 | 0.042 |
| TwinsUK | 2,006 | 0.42 | 0.98 | -0.025 | 0.53 | -0.047 | 0.48 | 0.32 |

AGES, Age, Gene/Environment Susceptibility; ARIC, Atherosclerosis Risk in Communities; B58C, British 1958 Cohort; CARDIA, Coronary Artery Risk Development in Young Adults; CHS, Cardiovascular Health Study; ECRHS, European Community Respiratory Health Survey; EPIC, European Prospective Investigation into Cancer and Nutrition; FEV_1_, forced expiratory volume in the first second; FHS, Framingham Heart Study; Health ABC, Health, Aging, and Body Composition Study; INT, interaction; MESA, Multi-Ethnic Study of Atherosclerosis; NA, not available; NFBC1966, Northern Finland Birth Cohort of 1966; RS, Rotterdam Study (cohorts I-III); SAPALDIA, Swiss Study on Air Pollution and Lung Diseases in Adults; SHIP, Study of Health in Pomerania; SNP, single nucleotide polymorphism.

^1^The joint *P* value corresponds to the 2 degrees-of-freedom joint test of the SNP main and interactive effect by Kraft et al. [[1](#_ENREF_1)]. The comparable 2 degrees-of-freedom joint test by Manning et al. [[2](#_ENREF_2)] is only applicable in the meta-analysis setting, as presented in the manuscript when combining results from all 19 studies.

**References**

1. Kraft P, Yen YC, Stram DO, Morrison J, Gauderman WJ (2007) Exploiting gene-environment interaction to detect genetic associations. Hum Hered 63: 111-119.

2. Manning AK, LaValley M, Liu C-T, Rice K, An P, et al. (2011) Meta-analysis of gene-environment interaction: joint estimation of SNP and SNPxEnvironment regression coefficients. Genet Epidemiol 35: 11-18.
