# Supplementary material for: Genome-Wide Joint Meta-Analysis of SNP and SNP-by-Smoking Interaction Identifies Novel Loci for Pulmonary Function
Source: PLoS Genet. 2012 Dec 20;8(12):e1003098. doi: 10.1371/journal.pgen.1003098 (PMC3527213; doi:10.1371/journal.pgen.1003098)
Supplement: Table S9 — Look-up evaluation of main SNP associations with cigarette smoking phenotypes using data generated by the Oxford-GlaxoSmithKline Consortium (N = 41,150), for the most significant SNP from each of the three novel loci implicated at genome-wide significance in the joint meta-analysis. (DOCX) [file pgen.1003098.s011.docx]

| **SNP**  **(coded allele)** | **Chr** | **Base pair position** | **Gene / closest gene(s)** | **Ever-smoking** | | | **Cigarettes per day** | | |
| --- | --- | --- | --- | --- | --- | --- | --- | --- | --- |
|  |  |  |  | **β** | **SE** | ***P*** | **β** | **SE** | ***P*** |
| rs7594321 (T) | 2q36.3 | 229,932,275 | *DNER* | -0.0066 | 0.010 | 0.53 | 0.0059 | 0.018 | 0.74 |
| rs7764819 (T) | 6p21.32 | 32,788,554 | *HLA-DQB1/ HLA-DQA2* | 0.0025 | 0.017 | 0.89 | 0.045 | 0.029 | 0.12 |
| rs11654749 (T) | 17q24.3 | 66,637,201 | *KCNJ2/ SOX9* | 0.018 | 0.010 | 0.072 | 0.0037 | 0.018 | 0.83 |

FEV_1_, forced expiratory volume in the first second; FVC, forced vital capacity; OR, odds ratio; SE, standard error; SNP, single nucleotide polymorphism.
