# Supplementary material for: Genome-Wide Joint Meta-Analysis of SNP and SNP-by-Smoking Interaction Identifies Novel Loci for Pulmonary Function
Source: PLoS Genet. 2012 Dec 20;8(12):e1003098. doi: 10.1371/journal.pgen.1003098 (PMC3527213; doi:10.1371/journal.pgen.1003098)
Supplement: Table S10 — mRNA expression profiling of three candidate genes in the human lung and periphery. Primer sequences are provided in Table S5. A “+” sign indicates presence of the transcript, and “−” indicates its absence. All products were sequence verified. (DOCX) [file pgen.1003098.s012.docx]

| **Gene** | **Human Tissue/Cells** | | | |
| --- | --- | --- | --- | --- |
|  | Lung | Airway smooth muscle | Bronchial epithilial cells | Peripheral blood mononuclear cells |
| *DNER* | + | + | + | + |
| *KCNJ2* | + | + | + | + |
| *SOX9* | + | + | + | - |
