# Supplementary material for: Genome-Wide Joint Meta-Analysis of SNP and SNP-by-Smoking Interaction Identifies Novel Loci for Pulmonary Function
Source: PLoS Genet. 2012 Dec 20;8(12):e1003098. doi: 10.1371/journal.pgen.1003098 (PMC3527213; doi:10.1371/journal.pgen.1003098)
Supplement: Table S11 — Questionnaire data used to ascertain cigarette smoking history (ever-smoking), amount, and duration across the 19 studies. Smoking amount and duration were used together to calculate pack-years. (DOCX) [file pgen.1003098.s013.docx]

| **Study** | **Question(s) and response categories used to ascertain smoking history** | ***For Ever-Smokers Only*** | |
| --- | --- | --- | --- |
|  |  | **Question(s) and response categories used to ascertain smoking amount (packs/day)** | **Question(s) and response categories used to ascertain duration (years smoked)** |
| AGES | Do you smoke cigarettes now? (Yes, No)  If you do not smoke cigarettes now, did you ever smoke cigarettes regularly? (Yes, No) | [Question for current smokers.] On average, how many cigarettes do you usually smoke per day? ( _ _ )  [Question for former smokers.] When you were smoking, about how many cigarettes did you smoke per day? ( _ _ ) | [Current & Former Smokers] About how old were you when you first started smoking cigarettes? ( _ _ )  [Former Smokers] About how old were you when you quit smoking cigarettes? ( _ _ ) |
| ARIC | Have you ever smoked cigarettes? (Yes, No)  (CODE "NO" IF LESS THAN 400 CIGARETTES IN A LIFETIME.) | On the average of the entire time you smoked, how many cigarettes did you usually smoke per day? | How old were you when you first started regular cigarette smoking? ( _ _ age)  Do you now smoke cigarettes? (Yes, No)  How old were you when you stopped?  ( _ _ age)  During the years that you have smoked. was there ever a period of one year or more that you did not smoke cigarettes? (Yes, No)  For how many years did you not smoke cigarettes? ( _ _ years) |
| B58C | Have you ever smoked cigarettes? (Yes/No [ascertained at ages 23, 33 and 42 years])  Positive responses included “occasional” smoking as well as “regular smoking” (>=1 cigarette per day) | How many cigarettes a day do you usually smoke? ( _ _ ) [ascertained at ages 23, 33, and 42 years] | No specific question was asked on age at starting smoking in the B58C.  Duration of regular cigarette smoking was therefore estimated from responses at ages 23, 33 and 42 years to questions on ever smoking and current smoking.  Duration of smoking, and therefore pack-years, were set to zero for lifelong non-smokers. |
| CARDIA | Do you still smoke cigarettes regularly? (Yes, No) | How many cigarettes do you smoke per day, on the average? ( _ _ cigarettes/day)  [If respondent has stopped smoking cigarettes:] How many cigarettes did you smoke per day, on the average before you stopped? ( _ _ cigarettes/day) | Altogether, how many years have you smoked cigarettes regularly? (Round Down).( _ _ years) |
| CHS | Have you smoked more than 100 cigarettes or 5 packs of cigarettes in your lifetime? (Yes, No, Don’t Know) | On the average of the entire time you smoked, how many cigarettes did you smoke per day?  (_ _ cigarettes per day) | Have you smoked cigarettes during the last 30 days? (Yes, No, Don’t know)  How old were you when you first started to smoke cigarettes? ( _ _ age in years)  If you have stopped smoking cigarettes completely, how old were you when you stopped? ( _ _ age in years) |
| ECRHS | Have you ever smoked for as long as a year? (Yes, No)  ['YES' means at least 20 packs of cigarettes or 12 oz (360 grams) of tobacco in a lifetime, or at least one cigarette per day or one cigara week for one year] | Do you now smoke, as of one month ago? (Yes, No)  [If yes,] how much do you now smoke on average? ( _ _ number of cigarettes, cigarillos, or cigars a week or pipe tobacco ounces/grams per week)  We calculated the total smoke pack per day assuming one smoke pack was equivalent to 20 cigarettes or 6.67 cigarillo or 20 grams of pipe tobacco or 4 cigars. | How old were you when you started smoking? ( _ _ years)  Do you now smoke, as of one month ago? (Yes, No)  If NO, How old were you when you stopped or cut down smoking? ( _ _ years)  Have you stopped or cut down smoking? (Yes, No)  If Yes, How old were you when you stopped or cut down smoking? ( _ _ years) |
| EPIC (obese cases and population-based) | Have you ever smoked as much as one cigarette a day for as long as a year? (Yes, No)  Do you smoke cigarettes now? (Yes, No) | [At each age 20, 30, 40, and 50], how many cigarettes did you smoke each day? ( _ _ cigarettes per day)  [For current smokers,] how many cigarettes do you smoke each day? ( _ _ ) | How old were you when you started smoking regularly? ( _ _ yrs old)  Do you smoke cigarettes now? (Yes, No)  If you have stopped smoking, how old were you when you gave up? ( _ _ yrs old) |
| FHS | Offspring cohort, examinations every 4-6 years starting in 1972  Have you smoked for at least one year? (Yes, smoke now / Yes, former smoker / No)  Have you smoked cigarettes? (Yes, smoke now / Yes, former smoker / No)  Follow-up examination  Have you smoked cigarettes regularly in the last year? (Yes, No)  Generation 3 cohort, initial examination starting in 2002  Have you ever smoked cigarettes regularly? (Yes, No)  [No means less than 20 pack of cigarettes or 20 oz. of tobacco in a lifetime or less than 1 cigarette a day for a day]  [If yes,] Have you smoked cigarettes regularly in the last year? (Yes, No) | Original cohort, biennial examinations starting in 1948  How many cigarettes per day do you smoke? ( _ _ )  Offspring cohort, examinations every 4-6 years starting in 1972  Usual number of cigarettes smoked ( _ _ cigarettes per day)  Follow-up examination  [For current smokers,] How many cigarettes do/did you smoke a day? ( _ _ )  Generation 3 cohort, initial examination starting in 2002  On the average of the entire time you smoked, how many cigarettes per day did you smoke? ( _ _ ) | Offspring cohort, examinations every 4-6 years starting in 1972  [For current and former smokers,] Age started smoking regularly ( _ _ )  If stopped, age stopped smoking ( _ _ )  Generation 3 cohort, initial examination starting in 2002  How old were you when you first started regular cigarette smoking? ( _ _ )  If you have stopped smoking cigarettes completely, how old were you when you stopped? ( _ _ ) |
| Health ABC | Have you smoked at least 100 cigarettes in your entire life? (Yes, No, Don’t know, Refused) | On the average of the entire time you smoked, how many cigarettes did you usually smoke per day? | How old were you when you first started smoking cigarettes fairly regularly? ( I was _ _ years old)  Do you smoke cigarettes now? (Yes, No)  How old were you when you stopped smoking? ( I was _ _ years old) |
| LifeLines | Have you ever smoked for at least a year? (Yes, No)  Do you smoke now, or have you smoked in the last month? (Yes, No)  Did you stop smoking? (Yes, No) | How much have you been smoking up till now?  From … yrs (age) till … yrs (age), I smoked … cigarettes/day  From … yrs (age) till … yrs (age), I smoked … cigarettes/day  From … yrs (age) till … yrs (age), I smoked … cigarettes/day  Etc. | How old were you when you started smoking?  ( _ _ yrs old)  How old were you when you stopped smoking?  ( _ _ yrs old)  How much have you been smoking up till now?  From … yrs (age) till … yrs (age), I smoked … cigarettes/day  From … yrs (age) till … yrs (age), I smoked … cigarettes/day  From … yrs (age) till … yrs (age), I smoked … cigarettes/day  Etc. |
| MESA | Have you ever smoked cigarettes? (Yes, No)  (“No” means less than 20 packs of cigarettes or 12 oz. of tobacco in your lifetime or less than 1 cigarette a day for one year at any time in your life.) | On the average of the entire time you smoked, how many cigarettes did you smoke per day? | How old were you when you first started smoking cigarettes? (age)  Have you smoked cigarettes during the last 30 days? (Yes, No)  How old were you when you quit smoking cigarettes? |
| NFBC1966 | Have you ever smoked in your life? (Yes, No)  (ascertained at the 31-year postal questionnaire) | How much per day do you usually smoke now or smoked before you gave up something? ( _ _ cigarettes per day [filter or other], pipefuls per day, or cigars per day)  (ascertained at the 31-year postal questionnaire)  We calculated the total smoke pack per day assuming one smoke pack was equivalent to 20 filter cigarettes or 10 other cigarettes or 8 pipefuls or 5 cigars. | I started smoking when I was __ years old  I have smoked regularly for altogether __ years (OR age at last smoke)  (ascertained at the 31-year postal questionnaire) |
| RS-I, RS-II, and RS-III | Do you currently smoke cigarettes? (Yes, No)  Did you smoke cigarettes in the past? (Yes, No) | How many cigarettes do you smoke a day?  ( _ _ number of cigarettes)  How many cigarettes did you smoke a day?  ( _ _ number of cigarettes) | How old were you when you started smoking cigarettes? ( _ _age)  How old were you when you stopped smoking cigarettes? ( _ _ age)  For how many years did you stop smoking (Past smokers)? ( _ _ years)  Did you ever stop smoking cigarettes (missing for current smokers)? (Yes, No)  For how many years did you stop smoking cigarettes (for current smokers)? ( _ _ years)  Number of smoking years ( _ _ years) |
| SAPALDIA | Have you ever smoked for as long as a year? (Yes, No)  (“Yes” means at least 20 packs of cigarettes or 12 oz (360 grams) of tobacco in a lifetime, or at least one cigarette per day or one cigar a week for one year)  Do you now smoke, as of one month ago? (Yes, No) | On average of the entire time you smoked, before you stopped, how much did you smoke? ( _ _ cigarettes per day)  How much do you now smoke on average? ( _ _ cigarettes per day) | How old were you when you started smoking? ( _ _ age)  How old were you when you stopped smoking? ( _ _ age) |
| SHIP | Do you smoke cigarettes now? (Yes, No)  Have you ever smoked cigarettes? (Yes, No) | How many cigarettes do you now smoke a day on average? ( _ _ cigarettes per day)  From when do you smoke this amount? (__ age)  What was the highest number of cigarettes you smoked daily over a period of one year? | How old were you when you started smoking? ( _ _ age)  How old were you when you started smoking? ( _ _ age)  When did you stop smoking? (__ age) |
| TwinsUK | Have you ever smoked a whole cigarette? (Yes, No)  Have you ever smoked cigarettes regularly (at least one cigarette per day at least 30 days)? (Yes, No)  In total have you smoked at least 100 cigarettes in your life? (Yes, No) | On the days that you smoked, how many cigarettes did you usually smoke? ( _ _ cigarettes per day)  During the years that you smoked daily, about how many cigarette per day did you usually smoke? ( _ _ cigarettes per day) | At what age did you start smoking? ( _ _ age)  At what age did you start smoking daily? ( _ _ age)  In your life, for how many years in total have you smoked daily? ( _ _ years)  How many cigarettes do you currently smoke? ( _ _ cigarettes per day)  How long is the longest period you have sucessfully quit smoking? ( _ _ days/months/years)  If you have stopped smoking, at what age did you stop altogether? ( _ _ age)  On how many of the last 30 days did you smoke at least one cigarette? ( _ _ days) |

AGES, Age, Gene/Environment Susceptibility; ARIC, Atherosclerosis Risk in Communities; B58C, British 1958 Cohort; CARDIA, Coronary Artery Risk Development in Young Adults; CHS, Cardiovascular Health Study; ECRHS, European Community Respiratory Health Survey; EPIC, European Prospective Investigation into Cancer and Nutrition; FEV_1_, forced expiratory volume in the first second; FVC, forced vital capacity; FHS, Framingham Heart Study; Health ABC, Health, Aging, and Body Composition Study; HWE, Hardy Weinberg equilibrium; MAF, minor allele frequency; MESA, Multi-Ethnic Study of Atherosclerosis; NFBC1966, Northern Finland Birth Cohort of 1966; RS, Rotterdam Study (cohorts I-III); SAPALDIA, Swiss Study on Air Pollution and Lung Diseases in Adults; SHIP, Study of Health in Pomerania; SNP, single nucleotide polymorphism.
