# Supplementary material for: Genome-Wide Joint Meta-Analysis of SNP and SNP-by-Smoking Interaction Identifies Novel Loci for Pulmonary Function
Source: PLoS Genet. 2012 Dec 20;8(12):e1003098. doi: 10.1371/journal.pgen.1003098 (PMC3527213; doi:10.1371/journal.pgen.1003098)
Supplement: Table S12 — Details of single nucleotide polymorphism (SNP) genotyping, quality control (QC), imputation, and statistical analysis across the 19 studies. (DOCX) [file pgen.1003098.s014.docx]

| **Study** | **Genotyping platform** | **QC filters for excluding genotyped SNPs** | **N, genotyped autosomal SNPs passing QC** | **Imputation software** | **NCBI Build for imputation reference (HapMap CEU)** | **N, SNPs used for analysis (MAF>3%)** | **Statistical analysis software** |
| --- | --- | --- | --- | --- | --- | --- | --- |
| AGES | Illumina HumanHap 370CNV | call rate<95%,  HWE P<10^-5^, or  MAF<1% | 326,034 | MACH v1.0.15 [[1](#_ENREF_1)] | build 35, release 21 | 2,325,257 | ProbAbel [[2](#_ENREF_2)] |
| ARIC | Affymetrix 6.0 | call rate<95%,  HWE P<10^-6^,  MAF<1%, or  no chromosomal location | 669,450 | MACH v1.0.16 [[1](#_ENREF_1)] | build 36, release 22 | 2,322,494 | ProbAbel [[2](#_ENREF_2)] |
| B58C^1^ | Illumina 550K (2 deposits) + 610K | call rate<95%,  HWE P<10^-4^,  MAF<1%, or  inconsistent (P<10^-4^) allele frequencies across 3 genotype deposits | 519,040 | MACH v1.0.16 [[1](#_ENREF_1)] | build 35, release 21 | 2,327,250 | ProbAbel [[2](#_ENREF_2)] |
| CARDIA | Affymetrix 6.0 | call rate<95%,  HWE P<10^-4^, or  MAF<2% | 578,568 | BEAGLE [[3](#_ENREF_3)] | build 36, release 22 | 2,287,974 | ProbAbel [[2](#_ENREF_2)] |
| CHS | Illumina HumanHap 370CNV | call rate<97%,  no heterozygotes,  HWE P<10^-5^,  >2 duplicate errors,  Mendelian inconsistency  (for HapMap CEU trios), or  no mapping in dbSNP | 306,655 | BIMBAM [[4](#_ENREF_4)] | build 36, release 22 | 2,281,530 | R [[5](#_ENREF_5)] |
| ECRHS | Illumina 610k | None | 582,892 | MACH 1.0 [[1](#_ENREF_1)] | build 36, release 22 | 2,337,606 | ProbAbel [[2](#_ENREF_2)] |
| EPIC obese cases | Affymetrix 500K | call rate < 90%,  HWE P <10^-6^, or  MAF<1% | 397,438 | IMPUTE v0.3.1 [[6](#_ENREF_6)] | build 35, release 21 | 2,504,711 | SNPTEST [[7](#_ENREF_7)] |
| EPIC population-based | Affymetrix 500K | call rate < 90%,  HWE P <10^-6^, or  MAF<1% | 397,438 | IMPUTE v0.3.1 [[6](#_ENREF_6)] | build 35, release 21 | 2,505,397 | SNPTEST [[7](#_ENREF_7)] |
| FHS^2^ | Affymetrix 500K + 50K Human Gene Focused Panel | call rate<97%,  HWE P<10^-6^,  MAF<1%,  differential missingness related to genotype (mishap procedure in PLINK[[8](#_ENREF_8)]) with P<10^-9^,  Mendelian errors>100, or  absence from HapMap | 378,163 | MACH v1.0.15 [[1](#_ENREF_1)] | build 36, release 22 | 2,323,290 | R [[5](#_ENREF_5)] |
| Health ABC | Illumina Human1M-Duo | call rate > 95%,  HWE P<10^-6^, or  MAF > 1% | 914,263 | MACH [[1](#_ENREF_1)] | build 36, release 22 | 2,331,622 | R [[5](#_ENREF_5)] |
| LifeLines | Illumina CytoSNP v2.0 | call rate < 99%,  HWE P<10^-4^, or  MAF<1% | 247,151 | BEAGLE 3.2 [[3](#_ENREF_3)] | build 36, release 24 | 1,833,720 | Quicktest [[9](#_ENREF_9)] |
| MESA | Affymetrix 6.0 | call rate < 95% or  monomorphic SNPs | 897,981 | IMPUTE v2.1.0 [[6](#_ENREF_6)] | build 36, release 24 | 2,438,158 | ProbAbel [[2](#_ENREF_2)] |
| NFBC1966 | Illumina CNV 370 Duo | call rate < 95%  HWE P < 10^-4^  MAF < 1% | 328,007 | IMPUTE v1.0 [[6](#_ENREF_6)] | build 35, release 21 | 2,303,023 | Quicktest [[9](#_ENREF_9)] |
| RS-I | Illumina HumanHap 550K | call rate<98%,  HWE P<10^-6^, or  MAF<1% | 512,349 | MACH v1.0.15 [[1](#_ENREF_1)] | build 36, release 22 | 2,313,611 | ProbAbel [[2](#_ENREF_2)] |
| RS-II | Illumina HumanHap 550K+610K | call rate<98%,  HWE P<10^-6^, or  MAF<1% | 537,405 | MACH v1.0.16 [[1](#_ENREF_1)] | build 36, release 22 | 2,464,493 | ProbAbel [[2](#_ENREF_2)] |
| RS-III | Illumina Human 610 Quad arrays | call rate<98%,  HWE P<10^-6^, or  MAF<1% | 591,893 | MACH v1.0.16 [[1](#_ENREF_1)] | build 36, release 22 | 2,466,288 | ProbAbel [[2](#_ENREF_2)] |
| SAPALDIA | Illumina Human 610K quad | call rate<97%,  HWE P<10^-4^, or  MAF<5% | 582,892 | MACH v1.0.16 [[1](#_ENREF_1)] | build 36, release 22 | 2,336,125 | ProbAbel [[2](#_ENREF_2)] |
| SHIP | Affymetrix 6.0 | none | 869,224 | IMPUTE v0.5.0 [[6](#_ENREF_6)] | build 36, release 22 | 2,395,357 | SNPTEST [[7](#_ENREF_7)] |
| TwinsUK^3^ | Illumina HumanHap 300K, 610Q, or 1M | call rate<95% if MAF>5%, call rate<99% if 1%<MAF<5%,  HWE P<5.7x10^-7^, or MAF<1% | 541,828 | IMPUTE v0.5.0 [[6](#_ENREF_6)] | build 36, release 22 | 2,236,002 | ProbAbel [[2](#_ENREF_2)] |

AGES, Age, Gene/Environment Susceptibility; ARIC, Atherosclerosis Risk in Communities; B58C, British 1958 Cohort; CARDIA, Coronary Artery Risk Development in Young Adults; CHS, Cardiovascular Health Study; ECRHS, European Community Respiratory Health Survey; EPIC, European Prospective Investigation into Cancer and Nutrition; FEV_1_, forced expiratory volume in the first second; FVC, forced vital capacity; FHS, Framingham Heart Study; Health ABC, Health, Aging, and Body Composition Study; HWE, Hardy Weinberg equilibrium; MAF, minor allele frequency; MESA, Multi-Ethnic Study of Atherosclerosis; NFBC1966, Northern Finland Birth Cohort of 1966; RS, Rotterdam Study (cohorts I-III); SAPALDIA, Swiss Study on Air Pollution and Lung Diseases in Adults; SHIP, Study of Health in Pomerania; SNP, single nucleotide polymorphism.

^1^ Two original subsets of B58C were combined for these analyses, following a new phase of genotyping with a common platform.

^2^To account for relatedness among subjects, the linear regression models implemented in FHS used a robust variance method via generalized estimating equations where each extended pedigree is a cluster and an independent working correlation structure is used.

^3^To correct for the twin-based ascertainment, the linear regression models implemented in TwinsUK used the pair-wide kinship matrix available in ProbAbel [[2](#_ENREF_2)].

**References**

1. Li Y, Abecasis GR (2006) Mach 1.0: Rapid Haplotype Reconstruction and Missing Genotype Inference. Am J Hum Genet S79: 2290.

2. Aulchenko YS, Ripke S, Isaacs A, van Duijn CM (2007) GenABEL: an R library for genome-wide association analysis. Bioinformatics 23: 1294-1296.

3. Browning BL, Browning SR (2009) A unified approach to genotype imputation and haplotype-phase inference for large data sets of trios and unrelated individuals. Am J Hum Genet 84: 210-223.

4. Servin B, Stephens M (2007) Imputation-based analysis of association studies: candidate regions and quantitative traits. PLoS Genet 3: e114.

5. Team RDC (2007) R: A language and environment for statistical computing. Vienna, Austria: R Foundation for Statistical Computing.

6. Howie BN, Donnelly P, Marchini J (2009) A flexible and accurate genotype imputation method for the next generation of genome-wide association studies. PLoS Genet 5: e1000529.

7. Marchini J, Howie B, Myers S, McVean G, Donnelly P (2007) A new multipoint method for genome-wide association studies by imputation of genotypes. Nat Genet 39: 906-913.

8. Purcell S, Neale B, Todd-Brown K, Thomas L, Ferreira MA, et al. (2007) PLINK: a tool set for whole-genome association and population-based linkage analyses. Am J Hum Genet 81: 559-575.

9. Kutalik Z, Johnson T, Bochud M, Mooser V, Vollenweider P, et al. (2011) Methods for testing association between uncertain genotypes and quantitative traits. Biostatistics 12: 1-17.
