# Supplementary material for: Genome-Wide Joint Meta-Analysis of SNP and SNP-by-Smoking Interaction Identifies Novel Loci for Pulmonary Function
Source: PLoS Genet. 2012 Dec 20;8(12):e1003098. doi: 10.1371/journal.pgen.1003098 (PMC3527213; doi:10.1371/journal.pgen.1003098)
Supplement: Table S13 — Study participants of European descent and quality control (QC) across the 19 studies. Participants passing QC filters and having acceptable spirometry data and complete covariate data were included in the meta-analyses. (DOCX) [file pgen.1003098.s015.docx]

| **Study** | **QC filters for excluding participants** | **N, genotyped participants** | **N, genotyped participants passing QC** | **N, genotyped participants**  **passing QC with spirometry and covariate data** |
| --- | --- | --- | --- | --- |
| AGES | call rate<97%,  discordances with previous genotypes | 3,660 | 3,219 | 1,696 |
| ARIC | call rate<95%,  sex mismatch,  discordances with prior genotyping,  >8 SD for any of the first 10 principal components,  outlying average identity-by-state estimates, or first-degree relatives | 10,898 | 9,131 | 8,934 |
| B58C | call rate<98%,  related individuals,  outliers on principal component analysis | 6,502 | 6,491 | 4,605 |
| CARDIA | call rate < 95%,  sex mismatch, or  outliers on principal component analysis | 1,725 | 1,720 | 1,605 |
| CHS | call rate<95%,  non-European ancestry,  sex mismatch, or  discordance with prior genotyping | 3,980 | 3,291 | 3,140 |
| ECRHS | call rate<95%,  duplicates or cryptic relatedness  sex mismatch  high X chromosomal heterozygosity for males,  non-European ancestry | 2,199 | 1,719 | 1,573 |
| EPIC obese cases | call rate<90% | 1,284 | 1,135 | 1,084 |
| EPIC population-based | call rate<90% | 2,566 | 2,417 | 2,294 |
| FHS | call rate<97%,  heterozygosity>5 SD from the mean, or  excessive non-inheritance | 9,274 | 8,481 | 7,694 |
| Health ABC | call rate < 97%,  sex mismatch, or  cryptic relatedness | 1,794 | 1,661 | 1,472 |
| LifeLines | call rate < 95%,  sex mismatch,  duplicate samples,  1^st^ degree relatives,  ethnic outliers, or  chromosomal abnormalities | 3,924 | 3,367 | 2,616 |
| MESA^1^ | call rate < 95%,  heterozygosity > 53%,  sex mismatch,  sample duplicates, or  first- or second-degree relatives | 2,693 | 2,650 | 1,403 |
| NFBC1966 | Contaminated sample,  duplicates,  gender mismatch,  no phenotype,  cryptic relatedness, or  withdrew consent | 4,772 | 4,763 | 3,564 |
| RS-I^1^ | call rate<97.5%,  excess autosomal heterozygosity,  sex mismatch, or  outlying identity-by-state clustering estimates | 6,240 | 5,974 | 1,196 |
| RS-II^1^ | call rate<97.5%,  excess autosomal heterozygosity,  sex mismatch, or  outlying identity-by-state clustering estimates | 2,516 | 2,157 | 840 |
| RS-III^1^ | call rate<97.5%,  excess autosomal heterozygosity,  sex mismatch, or  outlying identity-by-state clustering estimates | 3,932 | 2,082 | 1,224 |
| SAPALDIA | call rate<97%,  cryptic relatedness,  sex mismatch or excess X chromosomal heterozygosity for males,  non-European ancestry (outliers in principal component analysis) | 1612 | 1457 | 1,333 |
| SHIP^1^ | call rate<92%,  sex mismatch,  duplicates by identity-by-state | 4,105 | 4,081 | 1,768 |
| TwinsUK^2^ | call rate<98%,  heterozygosity across all SNPs>2 SD from the sample mean,  evidence of non-European ancestry as assessed by PCA comparison with HapMap3 populations;  observed pairwise IBD probabilities suggestive of sample identity errors | 6,558 | 5,654 | 2,006 |

AGES, Age, Gene/Environment Susceptibility; ARIC, Atherosclerosis Risk in Communities; B58C, British 1958 Cohort; CARDIA, Coronary Artery Risk Development in Young Adults; CHS, Cardiovascular Health Study; ECRHS, European Community Respiratory Health Survey; EPIC, European Prospective Investigation into Cancer and Nutrition; FHS, Framingham Heart Study; Health ABC, Health, Aging, and Body Composition Study; MESA, Multi-Ethnic Study of Atherosclerosis; NFBC1966, Northern Finland Birth Cohort of 1966; QC, quality control; RS, Rotterdam Study (cohorts I-III); SAPALDIA, Swiss Study on Air Pollution and Lung Diseases in Adults; SD, standard deviation; SHIP, Study of Health in Pomerania.

^1^A subset of genotyped participants had spirometry data available.

^2^A subset of unrelated TwinsUK participants was randomly selected for inclusion in these meta-analyses.
