# Supplementary material for: Genome-Wide Joint Meta-Analysis of SNP and SNP-by-Smoking Interaction Identifies Novel Loci for Pulmonary Function
Source: PLoS Genet. 2012 Dec 20;8(12):e1003098. doi: 10.1371/journal.pgen.1003098 (PMC3527213; doi:10.1371/journal.pgen.1003098)
Supplement: Table S14 — Primers for mRNA expression profiling. (DOCX) [file pgen.1003098.s016.docx]

| **Gene** | **Primer Name** | **Sequence** | **Expected product size (bp)** |
| --- | --- | --- | --- |
| *DNER* | DNER F | CTGCCAGCTTGTTGCAGAT | 418 |
|  | DNER R | GCCGAATCTTGACTGAGGT |  |
| *KCNJ2* | KCNJ2 F | TGCTCCTGCGCCAGCAACA | 931 |
|  | KCNJ2 R | AGGAGCTGTGCTCGAACAT |  |
| *SOX9* | SOX9 F | GCAAGCTCTGGAGACTTCT | 450 |
|  | SOX9 R | CGAAGGTCTCGATGTTGGA |  |
