# Supplementary material for: Genome-Wide Joint Meta-Analysis of SNP and SNP-by-Smoking Interaction Identifies Novel Loci for Pulmonary Function
Source: PLoS Genet. 2012 Dec 20;8(12):e1003098. doi: 10.1371/journal.pgen.1003098 (PMC3527213; doi:10.1371/journal.pgen.1003098)
Supplement: Text S1 — Detailed explanation of joint meta-analysis significance levels, in relation to main and interactive significance. (DOCX) [file pgen.1003098.s017.docx]

We performed simulation studies to better understand the observed discrepancies in significance between the joint test and the main and interaction effects. The simulation results (not shown) suggest that in models including interaction effects, the coding of the interacting variables may strongly alter the significance of the main SNP effect and the interaction effect, but not the significance of the joint test. For example, when simulating a model that includes an interaction between a SNP coded additively [0;1;2] and a binary exposure (such as ever-smoking), the significance of the joint test (of the main SNP effect and interaction effect) was always higher than the significance of the main SNP effect and interaction effect separately, even in the presence of main genetic effect only or interaction effect only. This pattern was not observed when the exposure was normally distributed. Further, as acknowledged by Manning et al. [[1](#_ENREF_1)], a nonlinear interaction effect or the presence of a threshold effect for a continuous environmental factor might give inconsistent JMA results between models based on the continuous versus binary parameterization of the environmental variable. The reduced significance, when the two interacting variables are coded with binary or categorical variable with few categories, is likely to be due to an overestimation of the variances and covariance terms between the two estimates (main and interaction effects). The joint test recovers this loss of significance because it accounts for this covariance term and provides some explanation for the observed results, where the joint test was much more significant than the main effect and the interaction effect.

**Reference**

1. Manning AK, Hivert MF, Scott RA, Grimsby JL, Bouatia-Naji N, et al. (2012) A genome-wide approach accounting for body mass index identifies genetic variants influencing fasting glycemic traits and insulin resistance. Nat Genet 44: 659-669.
